# RNA-Interference-Mediated miR-122-Based Gene Regulation in Colon Cancer, a Structural In Silico Analysis

**DOI:** 10.3390/ijms232315257

**Published:** 2022-12-03

**Authors:** Harsha Ganesan, Suman K. Nandy, Antara Banerjee, Surajit Pathak, Hong Zhang, Xiao-Feng Sun

**Affiliations:** 1 Department of Medical Biotechnology, Faculty of Allied Health Sciences, Chettinad Academy of Research and Education, Chettinad Hospital and Research Institute, Kelambakkam, Chennai 603103, Tamil Nadu, India; 2 BioNEST Bioincubator Facility, North-Eastern Hill University, Tura Campus, Chasingre, Tura 793022, Meghalaya, India; 3 Department of Oncology and Department of Biomedical and Clinical Sciences, Linköping University, 581 83 Linköping, Sweden; 4 School of Medical Sciences, Faculty of Medicine and Health, Orebro University, 702 81 Örebro, Sweden

**Keywords:** miR-122, colorectal cancer, RNA interference, Argonaute protein, gene silencing, network biology, signaling pathways

## Abstract

The role of microRNA 122 (miR-122) in colorectal cancer (CRC) has not been widely investigated. In the current study, we aimed to identify the prominent gene and protein interactors of miR122 in CRC. Based on their binding affinity, these targets were chosen as candidate genes for the creation of miR122–mRNA duplexes. Following this, we examined the miRNA-mediated silencing mechanism using the gene-silencing complex protein Argonaute (AGO). Public databases, STRING, and GeneMANIA were utilized to identify major proteins and genes interacting with miR-122. DAVID, PANTHER, UniProt, FunRich, miRwalk, and KEGG were used for functional annotation, pathway enrichment, binding affinity analysis, and expression of genes in different stages of cancer. Three-dimensional duplexes of hub genes and miR-122 were created using the RNA composer, followed by molecular interaction analysis using molecular docking with the AGO protein. We analyzed, classified, and scrutinized 93 miR-122 interactors using various bioinformatic approaches. A total of 14 hub genes were categorized as major interactors of miR-122. The study confirmed the role of various experimentally documented miR-122 interactors such as *MTDH* (Q86UE4), *AKT1* (P31749), *PTPN1* (P18031), *MYC* (P01106), *GSK3B* (P49841), *RHOA* (P61586), and *PIK3CG* (P48736) and put forth several novel interactors, with *AKT3* (Q9Y243), *NCOR2* (Q9Y618), *PIK3R2* (O00459), *SMAD4* (P61586), and *TGFBR1* (P36897). Double-stranded RNA duplexes of the strongest interactors were found to exhibit higher binding affinity with AGO. In conclusions, the study has explored the role of miR-122 in CRC and has identified a closely related group of genes influencing the prognosis of CRC in multiple ways. Further, these genes prove to be targets of gene silencing through RNA interference and might serve as effective therapeutic targets in understanding and treating CRC.

## 1. Introduction

The discovery of non-coding RNAs and their function as disease regulators has shifted the outlook of viewing RNAs from mere expression mediators to a biomolecule that aids the evolution of complex organisms. The past decade has witnessed a constant rise in non-coding RNA research, including the widely studied microRNAs (miRNA), a group of non-coding gene expression regulators that inhibit protein biosynthesis by binding to specific mRNAs and initiating the cleavage of primary miRNA that in turn adheres to the RNA-induced silencing complex (RISC) for beginning the downstream processes [1,2]. This mechanism has contributed to the use of miRNAs as potential biomarkers in a plethora of diseases including cancer. miRNA–mRNA interaction analysis has led to biomarker discovery in various types of cancers including colorectal cancer (CRC) [3], breast [4], and bladder [5] just to name a few. Various tumor suppressor miRNAs such as miR-29, miR-30, miR-17-3p, and miR-92, and oncogenic miRNAs such as miR-21 and miR-95 have been correlated with CRC [6,7].

Serum and plasma miRNAs are regarded as promising biomarkers in the field of oncology [8]. One such miRNA is miR-122, a serum exosomal miRNA abundantly present in CRC patients [9,10]. The presence of genes and corresponding proteins interacting with miR-122 has also been reported in various cases of CRC. This includes *TRIM*29, involved in PI3K/AKT regulation, *Bcl-W*, and *CCNG1* involved in apoptosis and cell cycle regulation [11]. Other interactors of miR-122 in CRC include CDC25A containing the complementary sequence (5′CACACUCC3′) [12], the XIAP protein that induced oxaliplatin resistance and contains the miR-122 binding site CACUCCA [13], and ALDOA protein in the SW480 and SW620 cells [14]. Similarly, the gene expression profiling of HCT 116, SW480, and SW620 CRC cell lines exhibited higher concentrations of miR-122 interactors including *AEG-1 (MTDH), CDK8, AKT, PCNA, NFKB,* and *PI3K* [15]. Further, the qRT-PCR analysis in CRC patients by Maierthaler et al. 2017 has highlighted the role of miR-122 as a prognostic biomarker [16]. Argonaute (AGO) proteins are a class of proteins involved in post-transcriptional regulation by the miRNA-mediated gene silencing process. Their central role in the RNA interference (RNAi) process begins by interacting and further cleaving the dsRNA complex in the RISC complex [17]. The approach of RNA-mediated gene regulation in CRC has not been studied in relation to miR-122. The mounting evidence of the mRNA and miRNA interaction has led to the speculation that miR-122 and its associated mRNAs could lead to substantial biomarker discovery in CRC. In the current study, we aimed to identify the key interactors of miR-122 in relation to CRC. We further aimed to analyze gene silencing by the mediation of the miR-122–mRNA complex.

## 2. Results

### 2.1. miRNA-122-Associated Genes

**Figure 1:**
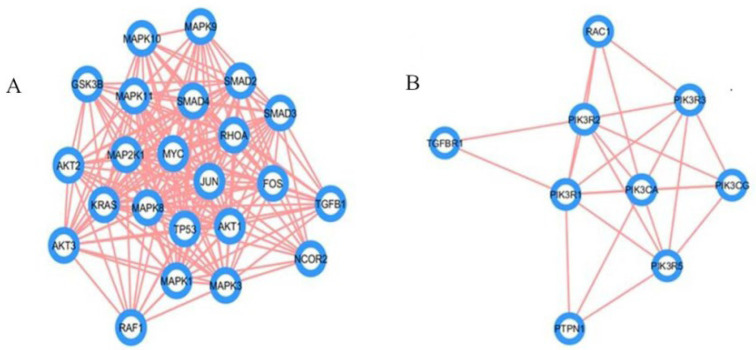
MCODE clustering of miR-122 interactors. (**A**) Largest cluster with twenty-three genes; (**B**) Second largest cluster with nine genes.

The screening of miRNA-122 interactors through miRNet 2.0 accessed knowledgebases such as miRTarBase, miR2Disease, TarBase, EpimiR, miRecords, HMDD, SM2miR, PharmacomiR, miRanda, starBase, and PhenomiR, and a total of 2152 (Table S4) genes and transcription factors were identified primarily and further curated by the network tool “Degree filter” using the InfoMap algorithm [18], and a total of 126 genes and transcription factors were retained [19]. Interaction data of the refined list of interactors was extracted utilizing the STRING and GeneMANIA apps of the Cytoscape software (version 3.8.0). A total of 13 genes were excluded as they were not detected in community detection using the InfoMap algorithm; hence, a total of 93 genes were secured in the final dataset (Table S1). Among the 93 interactors, 47 genes were solely identified through STRING, 30 genes exclusively by GeneMANIA, and 16 genes were common to both databases (Figure S1; Table S1). Following MCL network clustering using the cluster maker extension and the MCODE plug-in, two clusters with *p*-value ≤ 0.05 were identified comprising twenty-three and nine genes (Figure 1). The genes involved in the MAPK pathway such as MAPK1, MAPK3, and MAPK11 were found to have a higher degree of connectivity as exhibited by the MCODE analysis tool.

### 2.2. Functional Analysis and Pathway Enrichment Assessment

#### 2.2.1. Functional Classification

**Figure 2:**
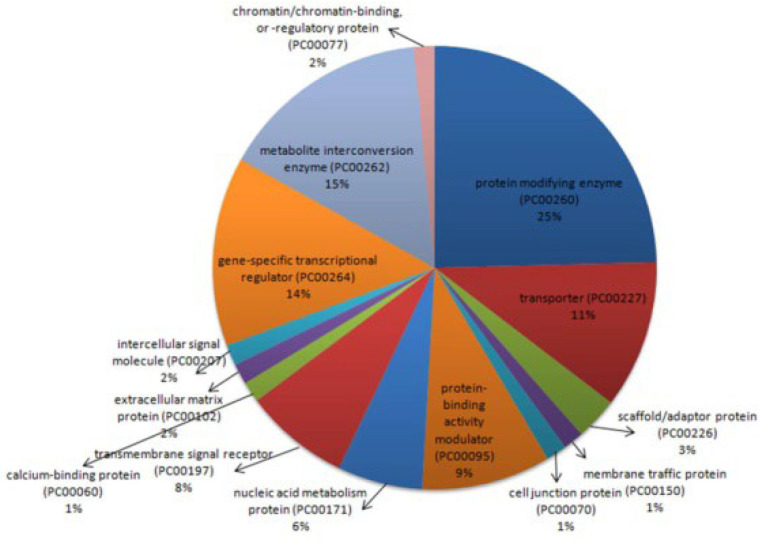
Protein class of miR-122 interactors identified using PANTHER classification system.

The 93 interactors of miR-122 were distributed over 14 protein classes according to PANTHER classification (Figure 2; Table S3). The majority of the proteins were classified under protein modification enzyme (25%), followed by metabolite interconversion enzyme (15%), gene-specific transcription regulator (14%), transporter (11%), protein-binding activity modulator (9%), transmembrane signal receptor (8%), and nucleic acid metabolism protein (6%).

#### 2.2.2. Functional Annotation

**Figure 3:**
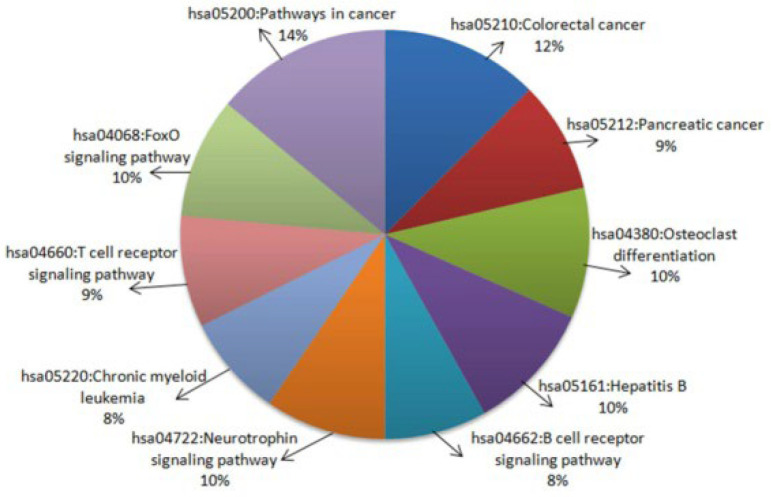
Pathways analyzed using the DAVID database.

**Figure 4:**
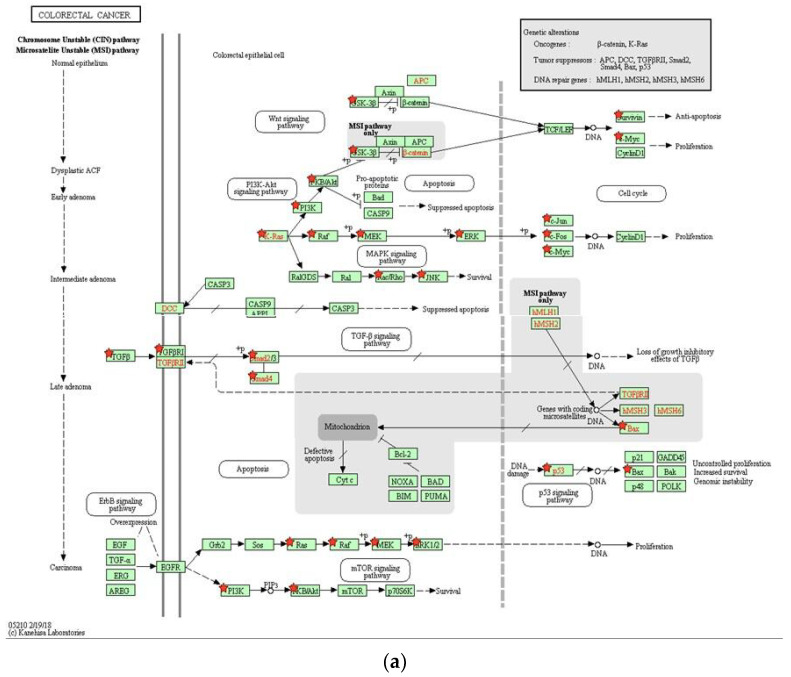

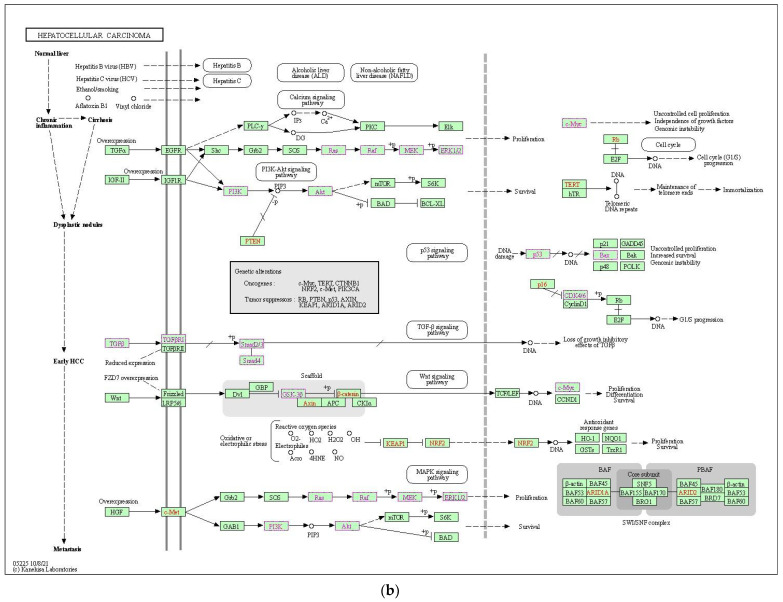

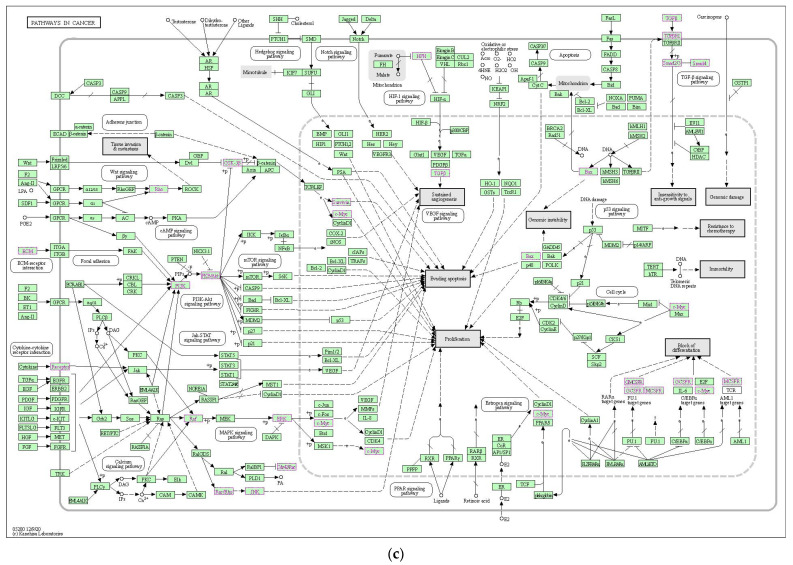
KEGG pathway analysis describing the presence of miR-122 interactors in different pathways; (**a**) DAVID KEGG colorectal cancer pathway analysis; (genes involved in the colorectal cancer pathway (red star) are target interactors of miR-122). (**b**) DAVID KEGG hepatocellular carcinoma pathway analysis; (miR-122 interactors involved in hepatocellular carcinoma are labelled in purple and red; purple: enhancers; red: inhibitors). (**c**) DAVID KEGG pathways in cancer analysis; (miR-122 interactors involved in major cancer-associated pathways are labelled in purple).

The DAVID Bioinformatics Resources 6.8 tool was used to analyze the major pathways involved in the miR-122 interactors. The top 10 KEGG pathways associated with the gene set were reported (Figure 3). The 35% of miR-122 interactors were recognized to be involved in different types of cancer pathways, namely, pathways in cancer (14%), colorectal cancer pathways (12%), and pancreatic cancer (9%). There were 3.75% of the interactors which were common to both pathways of cancer and CRC, namely, *PIK3CD*, *AKT1*, *AKT3*, *BAX*, *BIRC5*, *GSK3B*, *MAPK1*, *MYC*, *PIK3CG*, *PIK3R2*, *RAC1*, *RAF1*, *RHOA*, *SMAD2*, *SMAD4*, *TGFB1*, and *TGFBR1*. Figure 4a represents the KEGG pathway of colorectal cancer with the interactors of miR-122 marked. The analyses further demonstrate the involvement of the genes from different stages of CRC starting from colorectal epithelial cell progression to carcinoma. Major CRC genes including *PI3K, K-Ras*, and *Raf* present at the early adenoma stage of CRC, and *PKB/Akt* present at the late carcinoma stage is also part of the PI3K–Akt pathway. *GSK-3B* in the normal colon epithelial cells is a regulator of the Wnt signaling pathway. Other gene interactors in CRC such as *Ras*, *Raf*, *MEK,* at the early adenoma stage and *PKB/Akt* in the developed stage of CRC are also a part of the mTOR signaling pathway. Other pathways associated with the miR-122 included the p53 signaling pathway, MAPK signaling pathway, and the MSI pathway. Thus, confirming the significant role of miR-122 interactors at different stages of CRC progression and their association with numerous pathways as described in Figure 4b,c. Figure 4b depicts the involvement of *AKT1*, *MYC*, *TGFBR1, SMAD4*, and other miR-122 interactors also expressed in hepatocellular carcinoma. Figure 4c highlights the presence of several genes including *Rho*, *GSK3B*, *Ras*, *Raf, MEK, ERK*, *MYC*, *CDK4/6*, *TGFB*, and *SMAD4* in various other pathways of cancer. Hence, the affiliation is not restricted to CRC or hepatocellular carcinoma. Most of these genes such as KRAS, MYC, and PIK3R were also part of MCODE Cluster-1 (Figure 1A).

**Figure 5:**
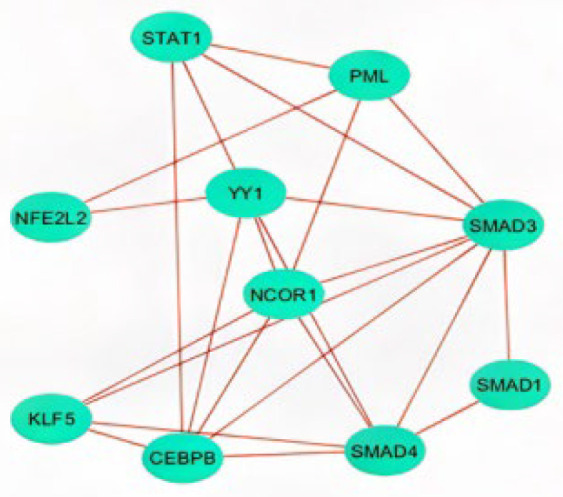
Interactome of top 10 transcription factors.

The involvement of transcription factors in controlling the interaction of miR-122-associated proteins was analyzed using the Enrichr tool. An adjusted *p*-value cut-off of 0.01–0.1 was used to filter the top 10 TF interactors and their possible interactivity was assessed. Through factor enrichment analysis, the interaction of the gene set with the top 10 transcription factors was examined, out of which CEBPB and NCOR1 had the highest number of edges (Figure S2). The transcription factors were scrutinized based on the higher number of interactions with miR-122 interactors. SMAD3, SMAD4, and CEBPB came up as key transcription factors regulating cancer progression at the late adenoma stage (Figure 4a). The tumor suppressor TFs SMAD3 and SMAD4 are involved in the TGF-ß signaling pathway regulation, and loss of which could lead to deregulation of apoptosis in the cell (Figure 4a; Table S3). To construct and analyze the interactome, the STRING database was used to study the interaction of the transcription factors with each other (Figure 5; Table S3). The closed interactome exhibited the mutual influence of the transcription factors.

#### 2.2.3. Functional Enrichment

**Figure 6:**
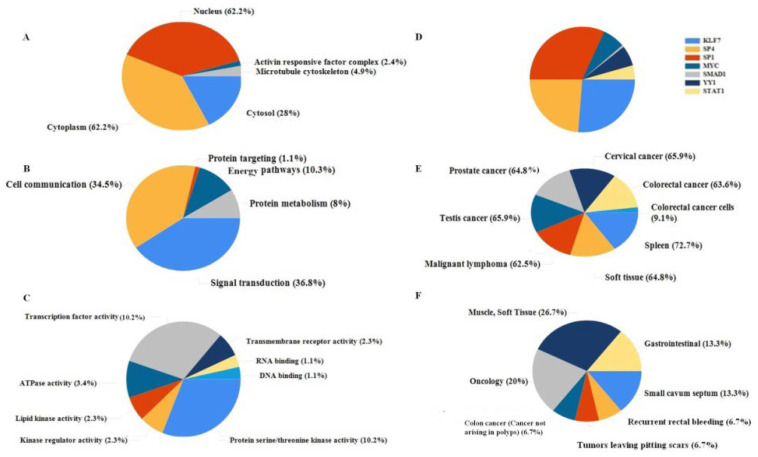
Functional enrichment of miR-122 interactors. (**A**) Subcellular localization of the interactors; (**B**) Involvement of interactors in the different biological process; (**C**) Interactor involvement in molecular function; (**D**) Transcription factor; (**E**) Site of expression; (**F**) Clinical phenotype.

Functional enrichment of the miR-122 interactors was carried out using the FunRich tool, and the gene ontology (GO) analysis consisted of six categories including cellular component (CC), biological process (BP), molecular function (MF), transcription factor (TF), site of expression (SE), and clinical phenotype (CP). The CC analysis (Figure 6A) exhibited that most proteins were classified as nuclear and cytoplasmic with a substantial amount of protein with dual subcellular localization. The BP examination (Figure 6B) stated a preponderance of proteins involved in signal transduction (36.8%) and cell communication (34.5%). MF analysis (Figure 6C) revealed 10.2% of interactors were involved in transcription factor activity and a similar number in phosphorylation. Through TF analysis (Figure 6D), it was found that the majority of interactors were activated by KLF7, SP1, and SP4. Through SE analysis (Figure 6E), the majority of the interactors were found to be expressed in cancer sites, 63.6% of the interactors were found in colorectal cancer, as well as other types of cancers including cervical, prostate, and testicular cancer. In agreement with the previous results, the CP (Figure 6F) exhibited a 20% alliance with oncology.

### 2.3. Analysis of Hub Genes and Binding Targets

**Figure 7:**
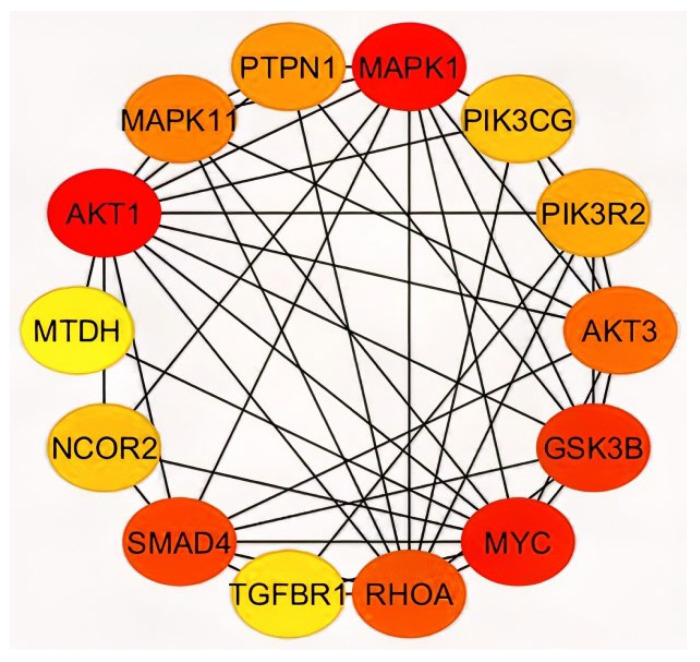
Major hub genes of miR-122; identification of hub genes through Cytohubba plugin in Cytoscape 3.8.0 software. The rank of connection degree is represented by the different degree of colors (from red to yellow).

With Cytohubba ranking by the maximal clique centrality (MCC), a total of 14 genes were scrutinized as Hub genes, this includes *AKT1*, *PIK3CG*, *PIK3R2*, *AKT3*, *GSK3B*, *MYC*, *RHOA*, *TGFBR1*, *SMAD4*, *NCOR2*, *MAPK1*, *MAPK11*, *PTPN1*, and *MTDH* (Figure 7; Table S3).

The subcellular localization of the hub genes revealed the majority of proteins including *AKT1*, *AKT3*, *GSK3B*, *MAPK1*, *MAPK11*, *MTDH*, *MYC*, *NCOR2*, *PIK3R2*, and *SMAD4* to be present at the nucleus (Table 1). Analysis of the site of expression revealed the existence of AKT1, MTDH, and NCOR2 genes in the colorectum and presence of TGFBR1 and MTDH in various cancer cell lines such as A-549 and HeLa (Table 1). These hub genes further exhibited a higher degree of interaction with each other and with miR-122. Following Cytohubba, the major interactors, region of interaction, and binding affinity were analyzed using miRwalk. The miRwalk database comprises miRNA prediction data from different databases including miRanda, miRDB, TargetScan, DIANA-mT, miRWalk, PICTAR4, PITA, RNAhybrid, PICTAR5, and RNA22 [20,21,22]. The hub genes exhibited 33 interaction sites with miR-122 (Table 2). It comprised 15 interaction sites in the coding region and 18 in the 3′ UTR region. The highest number of pairings (20) was observed in *GSK3B*. While the highest number of binding sites were present in *PIK3CG* and *PTPN1* as both exhibited five different binding sites in miR-122, followed by *MTDH* exhibiting four binding regions. Additionally, *GSK3B*, *NCOR2,* and *RHOA* displayed three binding sites for miR-122. The binding score is given on basis of a weighted scoring function consisting of binding region length, binding energy, and consecutive pairing. PIK3CG, MTDH, RHOA, and GSK3B had the highest binding score and were present in the 3′ UTR region. The longest consecutive pairing with 14 base pairs was observed in *PTPN1* and *SMAD4*.

### 2.4. Molecular Modelling of miRNA–mRNA Duplex

**Figure 8:**
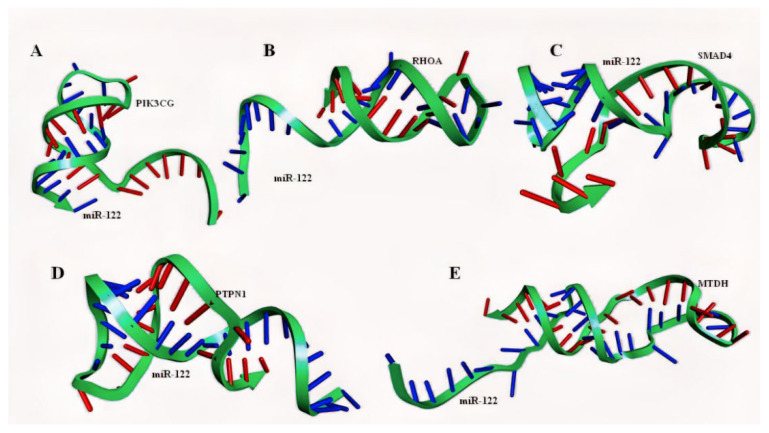
mRNA–miRNA duplexes; (blue: miRNA; red: mRNA of gene interactor) (**A**) miR-122–PIK3CG duplex; (**B**) miR-122–RHOA duplex; (**C**) miR-122–SMAD4 duplex; (**D**) miR122–PTPN1 duplex; (**E**) miR-122–MTDH duplex.

Duplex sequences between miR-122 and PIK3CG, RHOA, SMAD4, PTPN1, and MTDH were constructed using the RNA fold server, following the extraction of sequences from the miRwalk database and miRTarbase server. The secondary folding patterns of the duplexes, retrieved in the form of dot brackets were provided as inputs for the three-dimensional RNA duplex modeling using RNA composer (Figure 8). The crystallized AGO protein structure with 685 amino acids (PDB ID: 3F73) was extracted and modeled on Discovery Studio Visualizer. Following the deletion of water molecules and ligands, the A chain of this homo dimer was isolated and refined for further docking studies.

### 2.5. Molecular Docking of miRNA–mRNA–AGO Protein Complex

**Figure 9:**
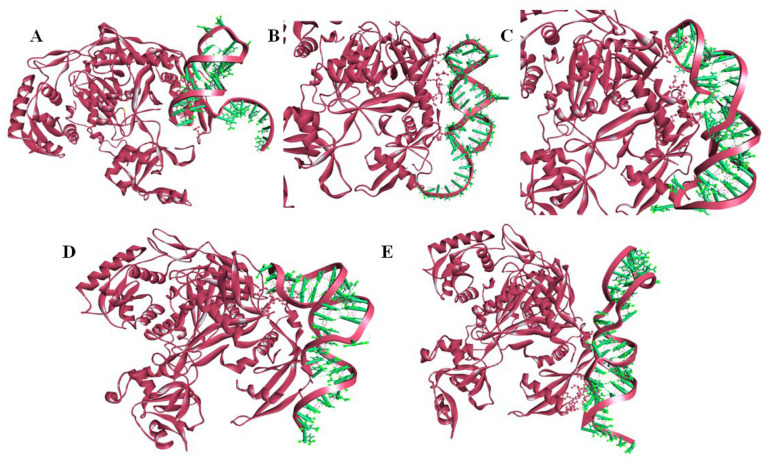
Molecular Docking of mRNA–miRNA duplexes and AGO protein; (**A**) miR-122–PIK3CG–AGO complex; (**B**) miR-122–RHOA–AGO complex; (**C**) miR-122–SMAD4–AGO complex; (**D**) miR122–PTPN1–AGO complex; (**E**) miR-122–MTDH–AGO complex.

A protein–nucleic-acid docking was carried out using the Haddock server and the pose with the highest geometric accuracy and atomic energy scores was scrutinized by the algorithm. Molecular docking of five different duplexes (miR-122-PIK3CG; miR-122-RHOA; miR-122-SMAD4; miR-122-PTPN1; miR-122-MTDH) was performed with the AGO protein. The interaction of miR-122–PIK3CG with AGO exhibited a carbon–hydrogen bond at ARG173 and MET172, electrostatic bonds at ARG173 and GLU122. The miR-122–RHOA–AGO complex interacted with conventional hydrogen bonds at LYS39, ARG395: HE, ARG395:HH21, and ARG396:HH21, while the miR-122–SMAD4–AGO complex demonstrated hydrogen linkages at LYS39 and LYS212, electrostatic bonds at ASP48, and hydrophobic bonds at LEU210 and MET400. Further, the miR-122–PTPN1–AGO complex exhibited a strong hydrogen bond at LYS402 and LYS739, electrostatic bonds at GLU46, and a hydrophobic interaction at TRP211. Finally, the miR-122–MTDH–AGO complex exhibited hydrogen bonds at ARG90:H and ARG90:NH2, electrostatic interactions at MET400, and hydrophobic interactions at ILE86. The presence of hydrogen bonds in the protein–nucleic-acid interaction further highlights the role of the AGO protein in gene regulation through miRNA and in RNA interference (Figure 9).

## 3. Discussion

miR-122 has been regarded as a potential tumor suppressor miRNA in various types of cancers including gastric cancer, rectal cancer, hepatocellular carcinoma, gallbladder cancer, uveal melanoma, and breast cancer [23,24]. The function of miR-122 and its associated mRNAs such as TRIM29, Bcl-W, CCNG1, CDC25A, XIAP, and ALDOA are reportedly downregulated during cancer cell progression [11,12,13,14]. Other key interactors of miR-122 include AEG-1 (MTDH), PI3K, CDK6, and PCNA [15]. Though a range of comprehensive research has been carried out on miRNA–mRNA interactions, the miR-122–mRNA regulatory network associated with CRC is far from being completely understood. The reliance on carcinogenic and tumor suppressor proteins as interaction partners has driven this study [22,23]. The current study aimed to identify and classify major genes interacting with miR-122 and to analyze and categorize the resulting phenotype functions in CRC.

The mRNA interactors of miR-122 were primarily obtained from the mirNet database (Table S1), but the gene set was scrutinized using algorithms such as InfoMap and tools such as STRING and GeneMania, followed by clustering using MCL (Figure S1) and MCODE (Figure 1). Most databases, use the Bayesian classifier, text, data mining, and other algorithms to predict the protein–protein interaction algorithm, that raises the question on the reliability of these data. Hence, the experimentally validated data were given preference in this study. The known mRNA interactors of miR-122 include PKM2, AKT1, MYC, TGFBR1, and SMAD4 in hepatocellular carcinoma (Figure 4b) [25], JNK, AP1, and caspases 3/8 in irritable bowel disease [26], and AKT, PI3K, PCNA, MTDH [15], PI3K, TRIM29, Bcl-W, CCNG1 [11], and ALDO2 [14] in CRC. A comparative analysis of miR-122 interactors in different pathways describes the presence of several genes associated with CRC including SMAD4, TGFBR1, AKT, MYC, Rho, Ras, Raf, Bax, and CDK 4/6 to actively engage in hepatocellular carcinoma and other pathways of cancer (Figure 4a–c). Hence, these multifunctional metabolic biomarkers were considered for further examination. Through analysis of MCC using the Cytohubba plug-in, the identified hub genes (Figure 7) exhibited a higher level of interaction with miR-122; this comprises of earlier experimentally proven genes including MTDH, AKT, PTPN1 [15], GSK3B, MYC [27], RHOA [28], PIK3CG, and AKT1 [29], and MAPK signaling related genes [30]. The results also included previously unreported genes including AKT3, NCOR2, PIK3R2, SMAD4, and TGFBR1, from which AKT3 and PIK3R2 were present in the early adenoma stage and SMAD4 and TGFBR1 were present at the late adenoma stage (Figure 4a). These genes were involved in different stages of cancer development as analyzed using the KEGG pathway (Figure 4a), this includes SMAD4 and TGFBR1 at the late adenoma, and AKT3 and PIK3R2 at the carcinoma stage in CRC development (Figure 4a). The gene clusters obtained through MCODE exhibited the presence of major hub genes including MYC, MAPK11, SMAD4, RHOA, NCOR2, PIK3R2, and PIK3CG.

miR-122 has emerged as an anti-proliferative and apoptotic component in cancerous cells, given its association with the PI3K/Akt signaling pathway [31]. Confirming this notion, the biological process (Figure 6B) has exhibited that 51.9% of the CRC interactors were involved in signal transduction and 43% were involved in cellular communication. Through molecular function assessment, a higher concentration of transcription activity (12.5%) (Figure 6C) was observed in the interacting molecules. Proving this, the TF enrichment analysis also exhibited the expression of various TFs including SP1, SP4, YY1, STAT1, and MYC, a hub gene (Figure 6D). Further investigation of TFs using the Enrichr tool (Figure S2) revealed interaction of the miR-122 interactor with TFs including KLF5, CEBPB, SMAD4, SMAD1, SMAD3, NCOR1, YY1, NFE2L2, PML, and STAT1. A total of seven TFs were obtained in the Enrichr software (Figure 5) and ten TFs were obtained from the FunRich tool (Figure 6D). Though few TFs found through the Enrichr tool such as NCOR1 and PML were also detected in the FunRich TF analysis, the results were excluded, given that these TFs consisted of only <1% of the interacting TFs. Previous studies have stated the importance of various TFs in the progression of CRC; hence, the expression studies stated various TFs including HNF4a and HNF6 to be associated with miR-122 [32]. The major TFs analyzed in our study have also been reported earlier. This includes the SMAD proteins (SMAD 4, SMAD 7) [33], STAT1 [34], CEBPB [35], and YY1 [36]. STAT1 has a wide range of interactions with other TFs in the interactome, including PML, CEBPB, SMAD3, and YY1. While YY1 also interacts with other molecules including SMAD3, NCOR1, SMAD4, CEBPB, NFE2L2, and STAT1 (Figure 5). Most of the analyzed proteins fall under a range of topological locations in the cell, and exhibit protein compartmentalization; this could be the probable cause of the dual localization observed in the miR-122 interactors.

Biological pathways play a major role in the regulation of all cellular processes including cancer progression and metastasis. The molecular pathways identified in CRC include chromosomal instability pathways, CpG methylator phenotype pathways, and microsatellite instability (MSI). Each pathway has been associated with a series of genes that tend to either upregulate or downregulate at different stages of cancer [37]. The genes analyzed in the current study, including Bax and GSK-3B, were expressed in the MSI pathway (Figure 4a). Through analysis of the KEGG pathway, a total of 14% of miR-122 interactors were associated with pathways in cancer and 12% were involved in CRC (Figure 3). These pathways include APC/b-Catenin pathway which contains the APC tumor suppressor gene, GSK3B, and the b-catenin gene which also takes part in the Wnt signaling [38]. The pathway analysis further highlighted the K-Ras, an oncogene, Raf, MEK, and other miR-122 interactors at the intermediate adenoma stage. Further, the tumor suppressor genes, SMAD2, SMAD4, and BAX, were also involved in the late adenoma stage (Figure 4a). Hence, the miR-122 interactors have established a strong hold on CRC using the integration of various pathways.

In total, nine of the fourteen hub genes, i.e., AKT1, AKT3, GSK3B, MAPK1, MYC, PIK3CG, PIK3R2, SMAD4, and TGFBR1 were present in both the STRING and GeneMANIA databases, analysis of MCODE further classified six of the hub genes, AKT1, AKT3, MAPK1, MAPK11, RHOA, and SMAD4 to be present in cluster one (Figure 1A), the largest cluster, and four of the hub genes, PIK3CG, PIK3R2, PTPN1, and TGFBR1, were present in the second cluster (Figure 1B). These genes were also reported to be present at different stages of cancer progression as reported by the KEGG pathway (Figure 4a). The TGFBR1 gene has also been confirmed to be a major interactor with the key transcription factors interacting with miR-122 (Figure 5). The site of CRC-specific protein expression has ranged from the cell surface to the nucleus at different stages of cancer development [39] in line with the evaluation of the CRC pathway through DAVID (Figure 4a) which exhibited the expression of various miR-122 associated proteins at different stages of cancer progression commencing from the colorectal epithelial cell, dysplastic aberrant crypt foci, different stages of adenoma, till carcinoma development. The site of expression analysis (Figure 6E) through functional enrichment of the proteome also exhibited 80.5% of the proteins predominant at the colon and 75.6% associated with CRC. 

The topology of the CRC proteins spans from transmembrane proteins such as TMEM180 [40] to extracellular vesicular proteins such as ACTB and JUB [41]. The subcellular localization could be classified into eight categories including the nucleus, cytoplasm, lysosome, endoplasmic reticulum, plasma membrane, Golgi apparatus, mitochondria, and extracellular components [42]. While the functional enrichment of the CRC gene set confirmed a higher concentration of proteins at the nucleus, cytoplasm, and cytosol (Figure 6A). Further investigation of the hub genes confirmed the same, as ten of the fourteen genes (AKT1, AKT3, GSK3B, MAPK1, MAPK11, MTDH, MYC, NCOR2, PIK3R2, and SMAD4) were present at the nucleus, and six of the fourteen genes were present at the cytoplasm (Table 1). Since miR-122 has been predominant at the nucleus [42], there is a higher chance of interaction with the corresponding mRNAs of hub genes.

The AGO protein serves as a primary regulator of RNAi by modulation of the post-transcriptional gene expression [43]. In the current study, the AGO protein structure was retrieved from PDB (PDB ID: 3F73; model organism: *Thermus thermophilus* HB27) as the expression system was *Escherichia coli*, and was further modelled using Discovery Studio Visualizer 3.5 suite prior to molecular docking [44]. The ability of the AGO protein to cleave the mRNA’s passenger strand and regulate the RISC-induced RNAi has made it the candidate protein for analysis of miRNA-induced gene regulation [17]. The genes with the highest bonding affinity with miR-122 were PIK3CG, RHOA, SMAD4, PTPN1, and MTDH, as per the miRwalk database (Table 2 and Table S2). Following this, the dsRNA duplex was created with the mRNAs of these candidate genes and miR-122 (Figure 8). To predict the structural interaction between the protein and the nucleic acid duplex, molecular docking serves as an effective computational technique as proven by Ruth et al. in the case of solid tumor [44]. Following the analysis, the duplexes exhibited a high binding affinity with the AGO protein. The presence of hydrogen bonds between the protein and the duplex highlights the role of these candidate mRNAs in CRC suppression. Among the five miRNA–mRNA duplexes, the miR-122–RHOA duplex showed higher bonding affinity as it contains various H bonds interacting with the ARG protein. Further, SMAD4 and MTDH also exhibited a high degree of interaction considering that they possess hydrogen, electrostatic, as well as hydrophobic bonds.

miRNA has been reported to bind to various regions of the target mRNA including the 3’ UTR, the TATA box, and other coding regions. The 3´ UTR region serves as a major target of the miRNA to induce the post-transcriptional gene silencing in the corresponding mRNA [45]. Hence, analysis of binding targets, especially the sites on the 3′ UTR, is necessary for predicting the possible fate of the protein. We identified a total of 33 binding regions of miR-122 in the hub genes (Table 2), out of which 18 sites were 3′ UTR regions, hence leading to possible mRNA silencing in various oncogenes. Amidst the eighteen binding sites, five sites were present in PIK3CG and four in MTDH and PTPN1 genes, respectively. Other genes containing the binding sites include *AKT1*, *AKT3*, *GSK3B*, *MAPK1*, *MAPK11*, *MYC*, *NCOR2*, *PIK3R2*, *RHOA*, *SMAD4*, and *TGFBR1* (Table 2). The *PIK3CG* gene would affect the MAPK signaling pathway. Inhibition of *MTDH* might lead to alteration in RNA binding activity and transcription regulation, and inhibition of the protein tyrosine phosphatase regulating protein, *PTPN1*, would further affect the JAK–STAT signaling pathway.

## 4. Materials and Methods

### 4.1. Prediction of miRNA Interactors

The list of possible interactors of the microRNA-122, namely, genes and transcription factors (TF) along with their immediate interactors using experimentally validated STRING entries, were screened, and enriched from miRNet 2.0 (http://www.mirnet.ca/ (accessed on 5 Febraury 2021)), a web-based tool for miRNA centric network building and analyses [19,46,47]. The curated gene set of major interactors of miR-122 was further validated through the published literature. Only the human interactors of miR-122 detected through experimental methods were retained. The gene and protein symbols were adopted as per the UniProtKB [48].

### 4.2. Functional Classification

PANTHER classification was employed to predict the protein class of miR-122 interacting genes [49,50]. Kyoto Encyclopedia of Genes and Genomes (KEGG) was used to determine the biological pathway association of miR-122 interactors [51]. Pathway enrichment was performed by DAVID 6.8 online tool [52] using KEGG pathways. The enriched ontology clusters were plotted based on the Kappa statistical similarities within the genes using the Metascape online platform (https://metascape.org/ (accessed on 5 October 2021)) [53,54]. The gene set was finally imported into the FunRich software for functional enrichment analysis, considering the protein domain, transcription factor, site of expression, clinical phenotype, cellular component, biological process, molecular function, and biological pathway association [55].

### 4.3. Network Clustering, Nodes, and Sub-Nodes Identification

A gene interaction network was visualized using the curated list of miR-122 interactors through Cytoscape version 3.8.0 using GeneMANIA (https://genemania.org/ (accessed on 10 February 2021)) [56,57] and a protein–protein interaction network using the STRING database (http://strin g-db.org (accessed on 10 February 2021)) [58]. The MCL algorithm was used to identify highly connected protein clusters using the ClusterMaker plug-in in Cytoscape [59]. Module identification, highly significant nodes, and sub-node classification was performed using the Cytoscape plug-in MCODE [60].

### 4.4. Enrichment Analysis of Transcription Factors

The Enrichr online tool was used to analyze the major transcription factors interacting with the gene set using the TRANSFAC_and_JASPER_PWMs category of the database. A statistically significant *p* value of ≤0.05 was maintained in all cases. The consortium of transcription factors and the interacting mRNAs were visualized using the Cytoscape 3.8.0 software and further, the interrelationship of transcription factors was predicted using STRING app in Cytoscape [61].

### 4.5. Analysis of Significant miR-122 Interactors

Hub genes from identified clusters and enriched pathways were plotted based on the degree of connectivity [62] through the Cytoscape plug-in Cytohubba. The Hub genes were further analyzed for the subcellular localization using the UniProt database, to analyse the miRNA–mRNA cellular topology [63].

### 4.6. Binding Site Analysis

The MiRWalk 2.0 database (http://mirwalk.umm.uni-heidelberg.de/ (accessed on 10 February 2021)), containing a repository of miRNA-target binding site analysis was used to identify the binding regions of miR-122 with the hub genes. The miR-122–mRNA interaction affinities were analyzed based on the binding site at 3’ UTR, coding sequence, and 5’ UTR regions, starting and ending regions of the binding targets, and binding site length [20].

### 4.7. Structural Prediction of miR122–mRNA Duplexes

The secondary structure of the miRNA and the target genes were designed using the RNAfold web server (http://rna.tbi.univie.ac.at/cgi-bin/RNAfold.cgi (accessed on 24 August 2022)) [64]. Following the folding affinity analysis, the dot brackets hence formed were provided as input data for the prediction of miR-122–mRNA duplex tertiary structures using the RNA COMPOSER tool (http://rnacomposer.cs.put poznan.pl/ (accessed on 24 August 2022)) [65].

### 4.8. Structural Extraction of Gene Regulator Protein

The AGO protein, the major gene regulator was extracted as a three-dimensional structure from Protein Data Bank (PDB ID: 3F73, Chain A). Further structural refinement was carried out in Discovery Studio Visualizer 3.5 suite [44,66].

### 4.9. Molecular Docking of miRNA–mRNA Duplex and AGO Protein

The protein–nucleic acid docking was carried out using the Haddock server. The RNA duplexes of the major interactors of miR-122 (miR-122 and AKT3; miR-122 and RHOA; miR-122 and SMAD4; miR-122 and PTPN1; miR-122 and MTDH) were selected based on the binding affinity scores and were docked with the AGO protein. The most prominent geometrical shape complementary score was produced by the Haddock algorithm. Further molecular interactions were analyzed using the Discovery Studio Visualizer 3.5 suite [44,67].

## 5. Conclusions

In the current study, we identified the diverse range of miR-122 interactors from several public databases. After further classification, we proceeded to analyze the protein class, subcellular localization, molecular function, biological function, cellular components, the associated pathways, and analysis of networks interacting with miR-122 and its interactors using a range of bioinformatics tools. The synergy of miR-122 interactors and the corresponding pathways was established. Further studying the subcellular localization of the major interactors and miR-122 confirmed their presence in the nucleus. Through analysis of the KEGG pathway, the miR-122 interactors chiefly fall under the categories “Pathways of Cancer” and “Colorectal Cancer”. In addition, the network analysis of the interacting proteins, genes, and transcription factors has led to scrutinization of fourteen hub genes that have a higher degree of interaction with miR-122. Five of these fourteen genes exhibited promising interaction sites with miR-122; hence, miRNA–mRNA duplexes were created with the genes and miR-122. These genes including PIK3CG, RHOA, SMAD4, PTPN1, and MTDH served as candidate genes for gene expression analysis through molecular docking analysis with the AGO protein. Hence, through the current research, we emphasize the role miR-122 bears as a major interactor and modulator of genes associated with CRC.

## Figures and Tables

**Table 1:** Cellular topology and site of expression of miR-122 interactors.

Gene Symbol	Uniprot ID	Tissue Specificity (Overexpressed Tissues)	Subcellular Location
AKT1	Q96B36	Liver, colon, and heart; high levels in cancer cell lines (A-549 and HeLa)	Nucleus, cytosol, and cytoplasm
AKT3	Q9Y243	Brain, lung, and kidney	Nucleus, cytosol, and cytoplasm
GSK3B	P49841	Testis, thymus, prostate, ovary, lungs, and intestine	Nucleus, plasma membrane, cytosol, and cytoplasm
MAPK1	P28482	Putamen, brain	Cytoplasm, cytosol, nucleus, and cytoskeleton
MAPK11	Q15759	Brain	Cytosol and nucleus
MTDH (AEG-1)	Q86UE4	Skeletal muscle, heart, small intestine, and in endocrine glands; Over expressed in various cancers including breast, brain, and colon cancer	Nucleus and endoplasmic reticulum
MYC	P01106	Thoracic mammary gland	Nucleus
NCOR2	Q9Y618	Colon, lung, spleen, and brain	Nucleus
PIK3CG	P48736	Pancreas, skeletal muscle, liver, and heart	Plasma membrane, cytoplasm, and cytosol
PIK3R2	O00459	Temporal lobe	Cytosol and nucleus
PTPN1	P18031	Keratinocytes	Endoplasmic reticulum
RHOA	P61586	Metanephric glomerulus	Cytoskeleton and plasma membrane
SMAD4	Q13485	Kidney	Nucleus, cytoplasm, and cytosol
TGFBR1	P36897	Abundant in most types of tissues; Also expressed in a variety of cancer cell lines	Plasma membrane

**Table 2:** Binding site prediction of miR122 and mRNA interactors.

Mirna ID	Refseq ID	Gene Symbol	Start	End	No. of Pairings	Binding Region Length	Binding Score	Binding Energy kcal/mol	Longest Consecutive Pairings	Position
hsa-miR-122-5p	NM_001282426	PIK3CG	5528	5549	19	21	1	−23	12	3UTR
hsa-miR-122-3p	NM_178812	MTDH	4741	4776	15	18	1	−21.9	13	3UTR
hsa-miR-122-5p	NM_001313943	RHOA	1063	1111	15	48	1	−21.3	7	3UTR
hsa-miR-122-5p	NM_178812	MTDH	3008	3026	15	18	1	−19.2	8	3UTR
hsa-miR-122-3p	NM_002093	GSK3B	4464	4501	12	14	1	−18.1	12	3UTR
hsa-miR-122-5p	NM_001278618	PTPN1	2530	2553	17	23	0.923	−20.6	7	3UTR
hsa-miR-122-5p	NM_001282426	PIK3CG	6288	6306	15	18	0.923	−19.5	9	3UTR
hsa-miR-122-5p	NM_178812	MTDH	4855	4872	14	17	0.923	−19.4	8	3UTR
hsa-miR-122-5p	NM_001278618	PTPN1	3077	3091	13	14	0.923	−18.7	13	3UTR
hsa-miR-122-3p	NM_001278618	PTPN1	2915	2930	13	15	0.923	−17.9	7	3UTR
hsa-miR-122-5p	NM_002751	MAPK11	1262	1281	15	19	0.846	−22	8	3UTR
hsa-miR-122-5p	NM_001282426	PIK3CG	5221	5244	19	23	0.846	−20.6	8	3UTR
hsa-miR-122-5p	NM_001282426	PIK3CG	3899	3916	14	17	0.846	−20	6	3UTR
hsa-miR-122-5p	NM_001278618	PTPN1	2459	2474	14	15	0.846	−19.7	14	3UTR
hsa-miR-122-3p	NM_178812	MTDH	4338	4352	13	14	0.846	−19.4	13	3UTR
hsa-miR-122-5p	NM_005359	SMAD4	3600	3653	19	33	0.846	−19.4	10	3UTR
hsa-miR-122-5p	NM_002467	MYC	2646	2666	17	20	0.846	−18.9	13	3UTR
hsa-miR-122-3p	NM_001282426	PIK3CG	3940	3967	14	17	0.846	−18.7	9	3UTR
hsa-miR-122b-3p	NM_001077261	NCOR2	2961	2976	13	15	0.961	−20.2	9	CDS
hsa-miR-122-5p	NM_181690	AKT3	990	1043	20	34	0.923	−24.5	8	CDS
hsa-miR-122-5p	NM_002093	GSK3B	1441	1474	20	33	0.923	-23.2	10	CDS
hsa-miR-122-5p	NM_004612	TGFBR1	1177	1201	19	24	0.846	−23.6	12	CDS
hsa-miR-122-5p	NM_001313943	RHOA	683	706	16	23	0.846	−19.8	8	CDS
hsa-miR-122-5p	NM_005359	SMAD4	1042	1061	17	19	0.846	−19.4	14	CDS
hsa-miR-122-5p	NM_001206654	NCOR2	596	642	16	21	0.846	−19.1	10	CDS
hsa-miR-122-5p	NM_001306210	TGFBR1	1189	1213	19	24	0.846	−18.6	12	CDS
hsa-miR-122b-3p	NM_005163	AKT1	1536	1553	15	17	0.846	−17.5	12	CDS
hsa-miR-122-5p	NM_138957	MAPK1	806	829	16	23	0.846	−17.2	6	CDS
hsa-miR-122b-5p	NM_005163	AKT1	1298	1323	15	20	0.846	−17	8	CDS
hsa-miR-122-5p	NM_001278618	PTPN1	677	701	19	24	0.846	−16.7	12	CDS
hsa-miR-122-5p	NM_001077261	NCOR2	622	642	16	20	0.846	−16.5	10	CDS
hsa-miR-122-3p	NM_002093	GSK3B	1779	1802	13	17	0.846	−16.3	11	CDS
hsa-miR-122-5p	NM_001313943	RHOA	382	402	18	20	0.846	−15.1	9	CDS

## Data Availability

On specific request to corresponding authors.

## Supplementary Materials

The supporting information can be downloaded at: https://www.mdpi.com/article/10.3390/ijms232315257/s1.
