# Supplementary material for: RNA-Interference-Mediated miR-122-Based Gene Regulation in Colon Cancer, a Structural In Silico Analysis
Source: Int J Mol Sci. 2022 Dec 3;23(23):15257. doi: 10.3390/ijms232315257 (PMC9739210; doi:10.3390/ijms232315257)
Supplement: Supplementary file 1 [file ijms-23-15257-s001.zip › ijms-1970768-supplementary.pdf]

Supplementary Material:

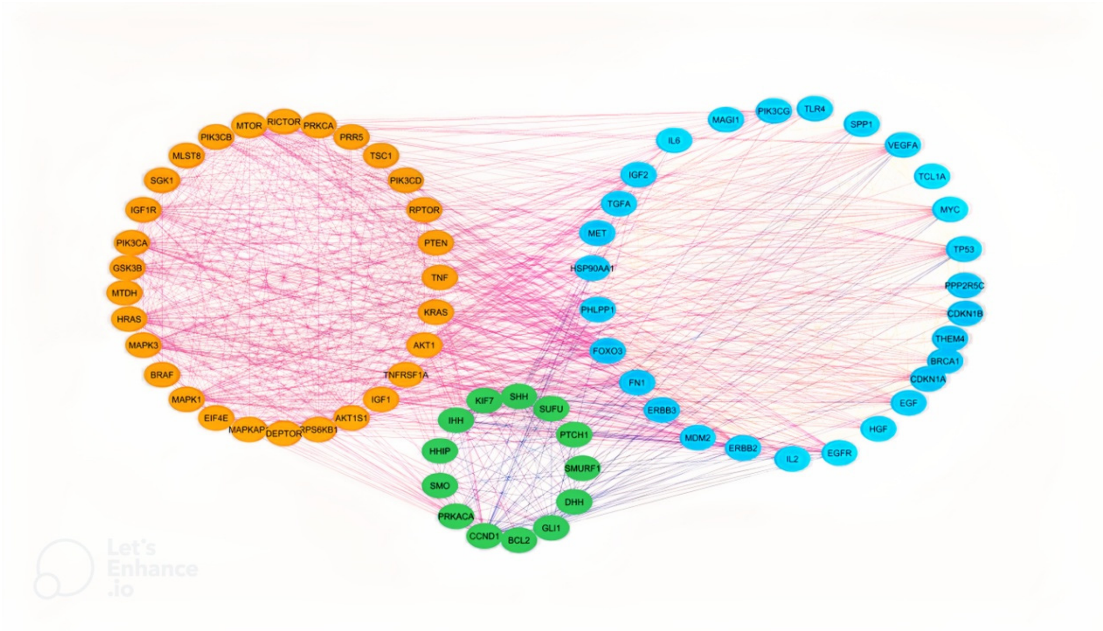

Figure S1. The integration of genes and proteins associated with miR-122 visualized by Cytoscape 3.8.0 software; (Orange, spherical nodes- Proteins obtained exclusively from STRING database; Blue, Spherical shaped nodes – Genes obtained solely from GeneMANIA; Green, spherical nodes – Common interactors obtained from both the databases).

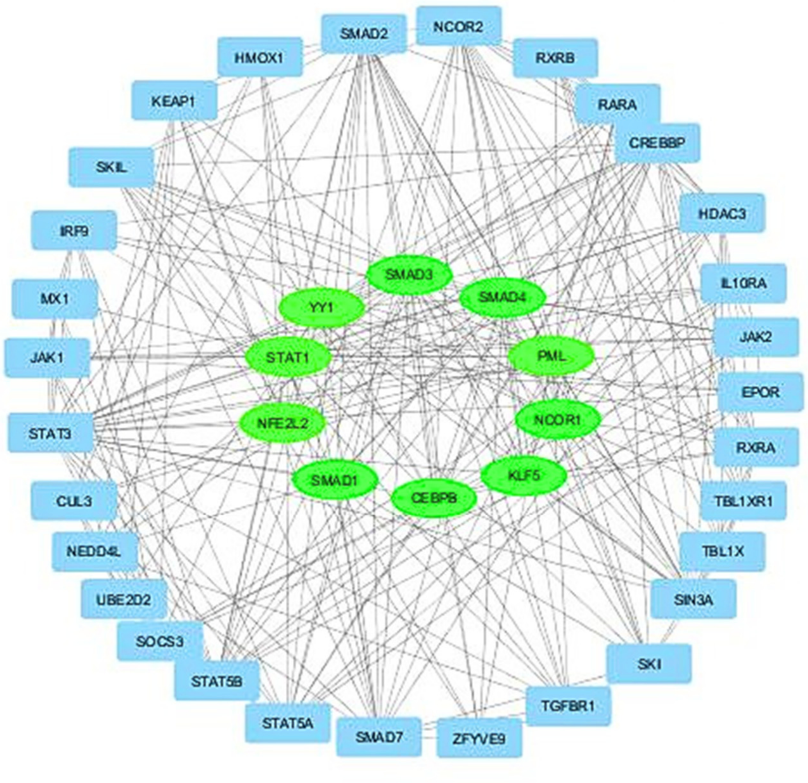

Figure S2. Predicted transcription factors of miR-122.

Table S1. Major interactors of miR-122

Gene name / Protein name	Gene symbol	UniProt ID	Interaction detection method	Source / Reference
Acetoacetyl-CoA Synthetase	*AACS*	Q86V21	Luciferase reporter assay, qRT-PCR	Mirtarbase, Tarbase
ABL Proto-Oncogene 2	*ABL2*	P42684	HITS-CLIP	Mirtarbase, Tarbase
A disintegrin and metalloprotease 17	*ADAM17*	P78536	Luciferase reporter assay, qRT-PCR	Mirtarbase, Tarbase
Aldolase, Fructose-Bisphosphate A	*ALDOA*	P04075	qRT-PCR, Dual-luciferase assay Northern blot	Li et al., 2019; Mirtarbase, Tarbase
Adaptor Related Protein Complex 3 Subunit Mu 2	*AP3M2*	P53677	Luciferase reporter assay, qRT-PCR	Mirtarbase, Tarbase
ATPase Na+/K+ Transporting Subunit Alpha 2)	*ATP1A2*	P50993	Luciferase reporter assay, qRT-PCR	Mirtarbase, Tarbase
BTB Domain And CNC Homolog 1	*BACH1*	O14867	HITS-CLIP	Tarbase
BTB Domain And CNC Homolog 2	*BACH2*	Q9BYV9	qRT-PCR	Mirtarbase, Tarbase
BCL2 Like 2	*BCL2L2*	Q92843	Immunohistochemistry, Luciferase reporter assay, Microarray, qRT-PCR, Reporter assay, Western blot	Mirtarbase, Tarbase
Cyclin G1	*CCNG1*	P51959	qRT-PCR,<br>qRT-PCR, Reporter assay, Western blot	Asadi et al., 2019,
Copper Chaperone For Superoxide Dismutase	*CCS*	O14618	Proteomics	Mirtarbase, Tarbase
Cyclin Dependent Kinase 6	*CDK6*	P49336	qRT-PCR	Malayaperumal et al., 2020
Claudin 18	*CLDN18*	P56856	qRT-PCR	Mirtarbase, Tarbase
Cut Like Homeobox 1	*CUTL1*	P39880	qRT-PCR	Mirtarbase, Tarbase
Dual Specificity Phosphatase 2	*DUSP2*	Q05923	Luciferase reporter assay, qRT-PCR	Mirtarbase, Tarbase
Ectonucleoside Triphosphate	*ENTPD4*	Q9Y227	Luciferase reporter assay, qRT-PCR	Mirtarbase, Tarbase

Diphosphohydrolase 4				
FA Complementation Group M	*FANCM*	Q8IYD8	Biotin-Microarrays	Tarbase
Forkhead Box J3	*FOXJ3*	Q8BUR3	Luciferase reporter assay, qRT-PCR	Mirtarbase, Tarbase
Forkhead Box P1	*FOXP1*	Q9H334	Luciferase reporter assay, qRT-PCR	Mirtarbase, Tarbase
FUN14 Domain Containing 2	*FUNDC2*	Q9BWH2	Luciferase reporter assay, qRT-PCR	Mirtarbase, Tarbase
Glucose-6-Phosphatase Catalytic Subunit 3	*G6PC3*	Q9BUM1	Luciferase reporter assay, Microarray, QRT-PCR	Mirtarbase, Tarbase
General Transcription Factor IIB	*GTF2B*	Q00403	qRT-PCR	Mirtarbase, Tarbase
Glycogen Synthase 1	*GYS1*	P13807	Microarray, Northern blot, qRT-PCR, Western blot	Mirtarbase, Tarbase
Interleukin 1 Receptor Type 1	*IL1R1*	P13504	HITS-CLIP	Tarbase
Interferon Regulatory Factor 9	*IRF9*	Q00978	HITS-CLIP	Tarbase
Laminin Subunit Gamma 1	*LAMC1*	P11047	Microarrays	Tarbase
Mitogen-Activated Protein Kinase Kinase Kinase 3	*MAP3K3*	Q99759	qRT-PCR	Mirtarbase, Tarbase
Mitogen-Activated Protein Kinase 11	*MAPK11*	Q15759	Luciferase reporter assay, qRT-PCR	Mirtarbase, Tarbase
methyl CpG binding protein 2	*MeCP2*	P51608	Luciferase reporter assay, qRT-PCR	Mirtarbase, Tarbase
Metadherin	*MTDH*	Q86UE4	qRT-PCR,<br>HITS-CLIP	Malayaperumal et al., 2020, Tarbase
Neural Cell Adhesion Molecule 1	*NCAM1*	P13591	Luciferase reporter assay, Microarray, qRT-PCR	Mirtarbase, Tarbase
Nuclear Receptor Corepressor 2	*NCOR2*	Q9Y618	Biotin-Microarrays	Tarbase
NDRG Family Member 3	*NDRG3*	Q9UGV2	Microarrays	Tarbase
Nuclear Factor Of Activated T Cells 1	*NFATC1*	O95644	qRT-PCR	Mirtarbase, Tarbase
Nuclear Factor Of Activated T Cells 2 Interacting Protein	*NFATC2IP*	Q8NCF5	Luciferase reporter assay, qRT-PCR	Mirtarbase, Tarbase
Nuclear Factor Kappa B	*NFKB*	P19838	qRT-PCR,	Malayaperumal et al.,

Subunit 1			Biotin-Microarrays	2020,<br>Mirtarbase, tarbase
NUMB Like Endocytic Adaptor Protein	*NUMBL*	Q9Y6R0	Luciferase reporter assay, qRT-PCR	Mirtarbase, Tarbase
ORAI Calcium Release-Activated Calcium Modulator 2	*ORAI2*	Q96SN7	HITS-CLIP, PAR-CLIP	Mirtarbase, Tarbase
Proliferating Cell Nuclear Antigen	*PCNA*	P12004	qRT-PCR	Malayaperumal et al., 2020
Phosphatidylinositol-4,5-Bisphosphate 3-Kinase Catalytic Subunit Alpha	*PI3K*	A0A2P0XJ53	qRT-PCR	Malayaperumal et al., 2020 ; Asadi et al., 2019
Protein Tyrosine Phosphatase Non-Receptor Type 1	*PTPN1*	P18031	qRT-PCR	Mirtarbase, Tarbase
RAD21 Cohesin Complex Component	*RAD21*	O60216	PAR-CLIP	Mirtarbase, Tarbase
Solute Carrier Family 7 Member 1	*SLC7A1*	P30825	Luciferase reporter assay, Northern blot, qRT-PCR, Reporter assay, Western blot	Mirtarbase, Tarbase
SMAD Family Member 2	*SMAD2*	Q15796	PAR-CLIP	Tarbase
Sequestosome 1	*SQSTM1*	Q13501	HITS-CLIP	Tarbase
TNF Receptor Superfamily Member 10b	*TNFRSF10B*	O14763	HITS-CLIP	Tarbase
Vav Guanine Nucleotide Exchange Factor 3	*VAV3*	Q9UKW4	Biotin-Microarrays	Tarbase
AKT Serine/Threonine Kinase 1	*AKT1*	P31749	qRT-PCR,<br>qPCR, Western Blot	Malayaperumal et al., 2020;<br>Tarbase
AKT Serine/Threonine Kinase 3	*AKT3*	Q9Y243	Immunoblot, Luciferase reporter assay, qRT-PCR	Mirtarbase, Tarbase
Bcl-2 Associated X-protein	*BAX*	Q07812	Microarray, Western blot	Mirtarbase, Tarbase
Baculoviral IAP Repeat Containing 5	*BIRC5*	O15392	qRT-PCR	Mirtarbase, Tarbase

Glycogen Synthase Kinase 3 Beta	*GSK3B*	P49841	HITS-CLIP	Mirtarbase, Tarbase
Mitogen-Activated Protein Kinase 1	*MAPK1*	P28482	qRT-PCR	Mirtarbase, Tarbase
MYC Proto-Oncogene	*MYC*	P01106	HITS-CLIP, Microarray	Mirtarbase, Tarbase
Phosphatidylinositol-4,5-Bisphosphate 3-Kinase Catalytic Subunit Delta	*PIK3CD*	O00329	Biotin-Microarrays	Tarbase
Phosphatidylinositol-4,5-Bisphosphate 3-Kinase Catalytic Subunit Gamma	*PIK3CG*	P48736	Luciferase Reporter Assay, qPCR, Western Blot	Tarbase
Phosphoinositide-3-Kinase Regulatory Subunit 2	*PIK3R2*	O00459	qRT-PCR	Mirtarbase, Tarbase
Rac Family Small GTPase 1	*RAC1*	P63000	Luciferase reporter assay, Microarray, qRT-PCR, Western blot	Mirtarbase, Tarbase
Raf-1 Proto-Oncogene	*RAF1*	P04049	Biotin-Microarrays	Tarbase
Ras Homolog Family Member A	*RHOA*	P61586	qRT-PCR	Mirtarbase, Tarbase
SMAD Family Member 4	*SMAD4*	Q13485	Biotin-Microarrays	Tarbase
Transforming Growth Factor Beta 1	*TGFB1*	P01137	qRT-PCR	Mirtarbase, Tarbase
Transforming Growth Factor Beta Receptor 1	*TGFBR1*	P36897	Biotin-Microarrays	Tarbase
AKT Serine/Threonine Kinase 2	*AKT2*	Q38898	qRT-PCR	Mirtarbase, Tarbase
Ankyrin 2	*ANK2*	Q01484	Luciferase reporter assay, qRT-PCR	Mirtarbase, Tarbase
Annexin A11	*ANXA11*	P50995	Luciferase reporter assay, Microarray, qRT-PCR	Mirtarbase, Tarbase
A-Raf Proto-Oncogene	*ARAF*	P10398	qRT-PCR	Mirtarbase, Tarbase
B-Raf Proto-Oncogene	*BRAF*	P15056	qRT-PCR	Mirtarbase, Tarbase
Chloride Intracellular Channel 4	*CLIC4*	Q9Y696	Luciferase reporter assay, Microarray, PAR-CLIP, qRT-PCR	Mirtarbase, Tarbase

CXADR Like Membrane Protein	*CLMP*	Q9H6B4	Microarrays	Mirtarbase, Tarbase
egl-9 family hypoxia inducible factor 3	*EGLN3*	Q9H6Z9	Luciferase reporter assay, qRT-PCR	Mirtarbase, Tarbase
Fos Proto-Oncogene	*FOS*	P01100	qRT-PCR	Mirtarbase, Tarbase
Jun Proto-Oncogene	*JUN*	P05412	qRT-PCR	Mirtarbase, Tarbase
Kirsten rat sarcoma virus	*KRAS*	P01116	qRT-PCR	Mirtarbase, Tarbase
Mitogen-Activated Protein Kinase 1	*MAP2K1*	Q02750	qRT-PCR	Mirtarbase, Tarbase
Mitogen-Activated Protein Kinase 12	*MAP3K12*	Q12852	qRT-PCR	Mirtarbase, Tarbase
Mitogen-Activated Protein Kinase 10	*MAPK10*	P53779	qRT-PCR	Mirtarbase, Tarbase
Mitogen-Activated Protein Kinase 3	*MAPK3*	P27361	qRT-PCR	Mirtarbase, Tarbase
Mitogen-Activated Protein Kinase 8	*MAPK8*	P45983	qRT-PCR	Mirtarbase, Tarbase
Mitogen-Activated Protein Kinase 9	*MAPK9*	P45984	qRT-PCR	Mirtarbase, Tarbase
Meprin A Subunit Alpha	*MEP1A*	Q16819	qRT-PCR	Mirtarbase, Tarbase
Phosphatidylinositol-4,5-Bisphosphate 3-Kinase Catalytic Subunit Alpha	*PIK3CA*	P42336	qRT-PCR	Mirtarbase, Tarbase
Phosphoinositide-3-Kinase Regulatory Subunit 1	*PIK3R1*	P27986	qRT-PCR	Mirtarbase, Tarbase
Phosphoinositide-3-Kinase Regulatory Subunit 3	*PIK3R3*	Q92569	qRT-PCR	Mirtarbase, Tarbase
Phosphoinositide-3-Kinase Regulatory Subunit 5	*PIK3R5*	Q8WYR1	qRT-PCR	Mirtarbase, Tarbase
RAB Interacting Factor	*RABIF*	P47224	qRT-PCR	Mirtarbase, Tarbase
Ras Association (RalGDS/AF-6) And Pleckstrin Homology	*RAPH1*	Q70E73	HITS-CLIP	Tarbase

Domains 1				
Solute Carrier Family 7 Member 11	*SLC7A11*	Q9UPY5	Luciferase reporter assay, qRT-PCR	Mirtarbase, Tarbase
SMAD Family Member 3	*SMAD3*	P84022	qRT-PCR	Mirtarbase, Tarbase
transforming growth factor beta 2	*TGFB2*	P61812	qRT-PCR	Mirtarbase, Tarbase
Tumor Protein P53	*TP53*	Q96S44	qRT-PCR	Mirtarbase, Tarbase
Tweety Family Member 3	*TTYH3*	Q9C0H2	qRT-PCR	Mirtarbase, Tarbase
Exportin 6	*XPO6*	Q96QU8	Luciferase reporter assay, qRT-PCR	Mirtarbase, Tarbase
ATPase Phospholipid Transporting 11A	*ATP11A*	P98196	qRT-PCR	Mirtarbase, Tarbase
B-cell lymphoma-W	*Bcl-W*	Q92843	qRT-PCR	Asadi et al., 2019
cell division cycle 25 homolog A	*CDC25A*	P30304	qRT-PCR, Dual-luciferase assay, Biotin-Microarrays	Yin et al., 2020 , Tarbase
GalNAc-T10; Polypeptide N-acetylgalactosaminyltransferase 10	*GALNT10*	Q86SR1	Immunohistochemistry, Immunoprecipitaion, In situ hybridization, Luciferase reporter assay, QRT-PCR, Western blot	Mirtarbase, Tarbase
RAB11 Family Interacting Protein 1	*RAB11FIP1*	Q6WKZ4	HITS-CLIP, Luciferase reporter assay, qRT-PCR	Mirtarbase, Tarbase
Ras-related protein Rab-6B	*RAB6B*	Q9NRW1	Luciferase reporter assay, qRT-PCR	Mirtarbase, Tarbase
Sterol O-Acyltransferase 1	*SOAT1*	P35610	CLEAR-CLIP, Microarrays	Tarbase
T-Box Transcription Factor 19	*TBX19*	O60806	Luciferase reporter assay, qRT-PCR	Mirtarbase, Tarbase
TPD52 Like 2	*TPD52L2*	O43399	Luciferase reporter assay, Microarray, qRT-PCR	Mirtarbase, Tarbase
Tribbles Pseudokinase 1	*TRIB1*	Q96RU8	Luciferase reporter assay, qRT-PCR	Mirtarbase, Tarbase
Tripartite Motif Containing 29	*TRIM29*	Q14134	qRT-PCR, Microarray	Asadi et al., 2019’ Mirtarbase, Tarbase
Ubiquitin Associated Protein 2	*UBAP2*	Q5T6F2	Luciferase reporter assay, qRT-PCR	Mirtarbase, Tarbase
X-linked inhibitor of apoptosis protein	*XIAP*	P98170	qRT-PCR, co-immunoprecipitation	Hua et al., 2018

Note: Gene and proteins from STRING database: Orange; GeneMania: Green; Yellow: Common in both STRING and GeneMania; Blue: Genes and proteins not included in the final dataset; Interactors indicated in blue were not shortlisted in the final dataset owing to their absence in either of the primary databases used for initial screening.

Table S2. Detailed binding site prediction of miR122 and mRNA interactors

Mirna id	Refseq id	Gene Name	Gene symbol	Start	End	No. of pairings	Binding region length	Binding score	Binding energy kcal/mol	Longest Consecutive pairings	Position
hsa-miR-122-5p	NM_001282426	Phosphatidylinositol-4,5-Bisphosphate 3-Kinase Catalytic Subunit Gamma	PIK3CG	5528	5549	19	21	1	-23	12	3UTR
hsa-miR-122-3p	NM_178812	Metadherin	MTDH	4741	4776	15	18	1	-21.9	13	3UTR
hsa-miR-122-5p	NM_001313943	Ras Homolog Family Member A	RHOA	1063	1111	15	48	1	-21.3	7	3UTR
hsa-miR-122-5p	NM_178812	Metadherin	MTDH	3008	3026	15	18	1	-19.2	8	3UTR
hsa-miR-122-3p	NM_002093	Glycogen Synthase Kinase 3 Beta	GSK3B	4464	4501	12	14	1	-18.1	12	3UTR
hsa-miR-122-5p	NM_001278618	Protein Tyrosine Phosphatase Non-Receptor Type 1	PTPN1	2530	2553	17	23	0.923	-20.6	7	3UTR
hsa-miR-122-5p	NM_001282426	Phosphatidylinositol-4,5-Bisphosphate 3-Kinase Catalytic	PIK3CG	6288	6306	15	18	0.923	-19.5	9	3UTR

		Subunit Gamma									
hsa-miR-122-5p	NM_178812	Metadherin	MTDH	4855	4872	14	17	0.923	-19.4	8	3UTR
hsa-miR-122-5p	NM_001278618	Protein Tyrosine Phosphatase Non-Receptor Type 1	PTPN1	3077	3091	13	14	0.923	-18.7	13	3UTR
hsa-miR-122-3p	NM_001278618	Protein Tyrosine Phosphatase Non-Receptor Type 1	PTPN1	2915	2930	13	15	0.923	-17.9	7	3UTR
hsa-miR-122-5p	NM_002751	Mitogen-Activated Protein Kinase 11	MAPK11	1262	1281	15	19	0.846	-22	8	3UTR
hsa-miR-122-5p	NM_001282426	Phosphatidylinositol-4,5-Bisphosphate 3-Kinase Catalytic Subunit Gamma	PIK3CG	5221	5244	19	23	0.846	-20.6	8	3UTR
hsa-miR-122-5p	NM_001282426	Phosphatidylinositol-4,5-Bisphosphate 3-Kinase Catalytic Subunit Gamma	PIK3CG	3899	3916	14	17	0.846	-20	6	3UTR
hsa-miR-122-5p	NM_001278618	Protein Tyrosine Phosphatase Non-Receptor Type 1	PTPN1	2459	2474	14	15	0.846	-19.7	14	3UTR
hsa-miR-122-3p	NM_178812	Metadherin	MTDH	4338	4352	13	14	0.846	-19.4	13	3UTR
hsa-miR-122-5p	NM_005359	SMAD Family Member 4	SMAD4	3600	3653	19	33	0.846	-19.4	10	3UTR
hsa-miR-122-5p	NM_002467	MYC Proto-Oncogene	MYC	2646	2666	17	20	0.846	-18.9	13	3UTR
hsa-miR-122-3p	NM_001282426	Phosphatidylinositol-4,5-Bisphosphate 3-	PIK3CG	3940	3967	14	17	0.846	-18.7	9	3UTR

		Kinase Catalytic Subunit Gamma									
hsa-miR-122b-3p	NM_001077261	Nuclear Receptor Corepressor 2	NCOR2	2961	2976	13	15	0.961	-20.2	9	CDS
hsa-miR-122-5p	NM_181690	AKT Serine/Threonine Kinase 3	AKT3	990	1043	20	34	0.923	-24.5	8	CDS
hsa-miR-122-5p	NM_002093	Glycogen Synthase Kinase 3 Beta	GSK3B	1441	1474	20	33	0.923	-23.2	10	CDS
hsa-miR-122-5p	NM_004612	Transforming Growth Factor Beta Receptor 1	TGFBR1	1177	1201	19	24	0.846	-23.6	12	CDS
hsa-miR-122-5p	NM_001313943	Ras Homolog Family Member A	RHOA	683	706	16	23	0.846	-19.8	8	CDS
hsa-miR-122-5p	NM_005359	SMAD Family Member 4	SMAD4	1042	1061	17	19	0.846	-19.4	14	CDS
hsa-miR-122-5p	NM_001206654	Nuclear Receptor Corepressor 2	NCOR2	596	642	16	21	0.846	-19.1	10	CDS
hsa-miR-122-5p	NM_001306210	Transforming Growth Factor Beta Receptor 1	TGFBR1	1189	1213	19	24	0.846	-18.6	12	CDS
hsa-miR-122b-3p	NM_005163	AKT Serine/Threonine Kinase 1	AKT1	1536	1553	15	17	0.846	-17.5	12	CDS
hsa-miR-122-5p	NM_138957	Mitogen-Activated Protein Kinase 1	MAPK1	806	829	16	23	0.846	-17.2	6	CDS
hsa-miR-122b-5p	NM_005163	AKT Serine/Threonine Kinase 1	AKT1	1298	1323	15	20	0.846	-17	8	CDS
hsa-miR-122-5p	NM_001278618	Protein Tyrosine Phosphatase Non-Receptor Type 1	PTPN1	677	701	19	24	0.846	-16.7	12	CDS

hsa-miR-122-5p	NM_001077261	Nuclear Receptor Corepressor 2	NCOR2	622	642	16	20	0.846	-16.5	10	CDS
hsa-miR-122-3p	NM_002093	Glycogen Synthase Kinase 3 Beta	GSK3B	1779	1802	13	17	0.846	-16.3	11	CDS
hsa-miR-122-5p	NM_001313943	Ras Homolog Family Member A	RHOA	382	402	18	20	0.846	-15.1	9	CDS

Table S3. Analysis of major miR-122 interactors

***in silico* tool used**	**Purpose**	**Results**		**Reference**
PANTHER	Determination of Protein classes	**Parameters Analysed**	**Percentage**	Figure 3
		Protein Modifying Enzyme	25%	
		Transporter	11%	
		Scaffold / Adaptor protein	3%	
		Membrane Traffic protein	1%	
		Cell Junction Protein	1%	
		Protein- binding activity modulator	9%	
		Nucleic Acid Metabolism Protein	6%	
		Transmembrane Signal Receptor	8%	
		Calcium-binding protein	1%	
		Extracellular matrix protein	2%	
		Intercellularsignal molecule	2%	
		Gene-specific transcriptional regulator	14%	
		Metabolite interconversion enzyme	15%	
		Chromatin/chromatin-binding or regulatory protein	2%	
DAVID 6.8 online tool	Determination of Overall pathways	**Parameters Analysed**	**Percentage**	Figure 4
		Colorectal cancer pathways	12%	

		Pancreatic cancer	9%	
		Osteoclast differentiation	10%	
		Hepatitis B	10%	
		B cell receptor signalling pathway	8%	
		Neurotrophin signalling pathway	10%	
		Chronic myeloid leukemia	8%	
		T cell receptor signalling pathway	9%	
		Fox O signalling pathway	10%	
		Pathways in cancer	14%	
DAVID – KEGG Pathway analysis	Identification of genes expressed in different stages of CRC pathway	Total number of genes from the geneset to be present in the KEGG pathway analysis is Twenty Eight. The genes were expressed in normal epithelium and in different stages if cancer progression including Dysplastic ACF, Early adenoma, intermediate adenoma, late adenoma, and carcinoma.		Figure 5
Enrichr	Identification of top 10 transcription factors in the gene set	STAT1, PML, YY1, NFE2L2, SMAD1, SMAD3, SMAD4, NCOR1, CEBPB, KLF5		Figure 6
FunRich	Functional Enrichment Analysis of gene set	**Parameters Analysed**	**Percentage**	Figure 7
		**A. Sub-cellular localization**		Figure 7a.
		Nucleus	62.2%	
		Activity responsive factor complex	2.4%	
		Microtubule cytoskeleton	4.9%	
		Cytosol	28%	
		Cytoplasm	62.2%	
		**B. Biological Process**		Figure 7b.
		Protein targeting	1.1%	
		Energy pathways	10.3%	
		Protein metabolism	8%	
		Signal transduction	36.8%	
		Cell communication	34.5%	

		**C. Molecular Function**		Figure 7c.
		Transcription factor activity	10.2%	
		Transmembrane receptor activity	2.3%	
		RNA binding	1.1%	
		DNA binding	1.1%	
		Protein serine/threonine kinase activity	10.2%	
		Kinase regulator activity	2.3%	
		Lipid kinase activity	2.3%	
		ATPase activity	3.4%	
		**D. Transcription factor**		Figure 7d.
		KLF7		
		SP4		
		SP1		
		MYC		
		SMAD1		
		YY1		
		STAT1		
		**E. Site of expression**		Figure 7e.
		Cervical cancer	65.9%	
		Colorectal cancer	63.6%	
		Colorectal cancer cells	9.1%	
		Spleen	72.7%	
		Soft tissue	64.8%	
		Malignant lymphoma	62.5%	
		Testis cancer	65.9%	
		Prostate cancer	64.8%	
		**F. Clinical Phenotype**		Figure 7f.
		Muscle, Soft tissue	26.7%	

		Gastrointestinal				13.3%		
		Small cavum septum				13.3%		
		Recurrent rectal bleeding				6.7%		
		Tumors leaving pitting scars				6.7%		
		Colon cancer				6.7%		
		Oncology				20%		
Cytoscape 3.8.0. software (Cytohubba plug-in)	Identification of major hub genes	AKT1, MAPK1, MYC, GSK3B, SMAD4, RHOA, AKT3, MAPK11, PTPN1, PIK3R2, NCOR2, TGFBR1, PIK3CG, MTDH						Figure 8
MiRWalk 2.0 database	Binding site prediction of miR122 and Hub genes (mRNA)	**Gene Symbol**	**Start**	**End**	**Binding region length**	**Binding energy kcal/mol**	**Position**	Table 2
		MTDH	4741	4776	18	-21.9	3UTR	
			3008	3026	18	-19.2	3UTR	
			4855	4872	17	-19.4	3UTR	
			4338	4352	14	-19.4	3UTR	
		PIK3CG	5528	5549	21	-23	3UTR	
			6288	6306	18	-19.5	3UTR	
			5221	5244	23	-20.6	3UTR	
			3899	3916	17	-20	3UTR	
			3940	3967	17	-18.7	3UTR	
		RHOA	1063	1111	48	-21.3	3UTR	
			683	706	23	-19.8	CDS	
			382	402	20	-15.1	CDS	
		GSK3B	4464	4501	14	-18.1	3UTR	
			1441	1474	33	-23.2	CDS	
			1779	1802	17	-16.3	CDS	
		PTPN1	2530	2553	23	-20.6	3UTR	

			3077	3091	14	-18.7	3UTR	
			2915	2930	15	-17.9	3UTR	
			2459	2474	15	-19.7	3UTR	
			677	701	24	-16.7	CDS	
		MAPK11	1262	1281	19	-22	3UTR	
			3600	3653	33	-19.4	3UTR	
		SMAD4	1042	1061	19	-19.4	CDS	
		MYC	2646	2666	20	-18.9	3UTR	
			2961	2976	15	-20.2	CDS	
			596	642	21	-19.1	CDS	
		NCOR2	622	642	20	-16.5	CDS	
		AKT3	990	1043	34	-24.5	CDS	
			1177	1201	24	-23.6	CDS	
		TGFBR1	1189	1213	24	-18.6	CDS	
		AKT1	1536	1553	17	-17.5	CDS	
			1298	1323	20	-17	CDS	
		MAPK1	806	829	23	-17.2	CDS	

Table S4. Complete list of miR122 interactors

No.	Gene Symbol	miRnet Target ID	Experiment used for detection	No.	Gene Symbol	miRnet Target ID	Experiment used for detection
1.	A2M	2	Microarray	1077	LPCAT3	10162	Biotin-Microarrays
2.	ABCF1	23	HITS-CLIP//PAR-CLIP	1078	NT5DC3	51559	Microarrays
3.	ABL2	27	HITS-CLIP	1079	SUDS3	64426	Biotin-Microarrays
4.	ADAM10	102	Immunohistochemistry//Luciferase reporter assay//qRT-PCR//Western blot	1080	FGFR1OP2	26127	Biotin-Microarrays
5.	ADCY2	108	HITS-CLIP	1081	RNGTT	8732	Biotin-Microarrays
6.	GRK3	157	HITS-CLIP	1082	NCOA7	135112	Biotin-Microarrays
7.	ALDOA	226	Luciferase reporter	1083	FBXO9	26268	Biotin-Microarrays

			assay//Northern blot//qRT-PCR				
8.	ALOX5AP	241	PAR-CLIP	1084	MDN1	23195	Biotin-Microarrays
9.	ANG	283	HITS-CLIP	1085	ZNF451	26036	Biotin-Microarrays
10.	ANK2	287	Luciferase reporter assay//qRT-PCR	1086	BAG2	9532	Biotin-Microarrays
11.	ANXA7	310	Proteomics	1087	E2F3	1871	Biotin-Microarrays
12.	ANXA11	311	Luciferase reporter assay//Microarray//qRT-PCR	1088	B3GAT2	135152	Biotin-Microarrays
13.	XIAP	331	HITS-CLIP	1089	SOBP	55084	Biotin-Microarrays
14.	BIRC5	332	PAR-CLIP	1090	TRIM38	10475	Biotin-Microarrays
15.	RHOA	387	Luciferase reporter assay	1091	PHF1	5252	Microarrays
16.	PHOX2A	401	Microarray	1092	QKI	9444	HITS-CLIP, Biotin-Microarrays
17.	ARL2	402	PAR-CLIP	1093	ENPP5	59084	Biotin-Microarrays
18.	ARSA	410	PAR-CLIP	1094	BRD8	10902	Biotin-Microarrays
19.	ARSB	411	Microarray	1095	LMNB1	4001	Microarrays
20.	ART3	419	Microarray	1096	LNPEP	4012	Biotin-Microarrays
21.	ATP1A2	477	Luciferase reporter assay//qRT-PCR	1097	TRAPPC13	80006	Biotin-Microarrays
22.	ATP7A	538	Microarray	1098	ATP6V0E1	8992	Biotin-Microarrays
23.	AXL	558	Luciferase reporter assay	1099	CPEB4	80315	Biotin-Microarrays
24.	BAX	581	Microarray//Western blot	1100	ZNF346	23567	Biotin-Microarrays
25.	BCL2L1	598	qRT-PCR//Western blot	1101	SMC4	10051	Biotin-Microarrays
26.	BCL2L2	599	Immunohistochemistry//Luciferase reporter assay//Microarray//qRT-PCR//Reporter assay//Western blot	1102	BCL6	604	Biotin-Microarrays
27.	BPGM	669	Microarray	1103	KPNA1	3836	HITS-CLIP
28.	BRCA2	675	Microarray	1104	ARMC8	25852	Biotin-Microarrays
29.	C3	718	HITS-CLIP	1105	SLC25A36	55186	Biotin-Microarrays
30.	CALD1	800	Proteomics	1106	COL7A1	1294	Biotin-Microarrays, Microarrays
31.	CALM3	808	PAR-CLIP	1107	PRKAR2A	5576	Biotin-Microarrays
32.	CALR	811	Proteomics	1108	USP4	7375	Biotin-Microarrays
33.	CALU	813	Proteomics	1109	TUSC2	11334	Microarrays

34.	CASP7	840	Microarray	1110	FXR1	8087	Biotin-Microarrays
35.	CCNG1	900	qRT-PCR//Reporter assay//Western blot	1111	BBX	56987	PAR-CLIP, Biotin-Microarrays
36.	SIGLEC6	946	PAR-CLIP	1112	PLXNA1	5361	Biotin-Microarrays
37.	ENTPD1	953	HITS-CLIP	1113	PLSCR4	57088	Microarrays
38.	CDK4	1019	HITS-CLIP//Reporter assay	1114	MAPKAPK3	7867	Biotin-Microarrays
39.	CENPF	1063	Proteomics	1115	ZBTB47	92999	Biotin-Microarrays
40.	CEACAM 8	1088	Microarray	1116	NKTR	4820	Biotin-Microarrays
41.	AP1S1	1174	PAR-CLIP	1117	NEK4	6787	Biotin-Microarrays
42.	CCR6	1235	HITS-CLIP	1118	INO80D	54891	Biotin-Microarrays
43.	CNN3	1266	Proteomics	1119	GTF3C2	2976	Microarrays
44.	COL13A1	1305	HITS-CLIP	1120	IFIH1	64135	Microarrays
45.	COPA	1314	PAR-CLIP	1121	SPTBN1	6711	Biotin-Microarrays
46.	SLC31A1	1317	HITS-CLIP	1122	USP34	9736	Biotin-Microarrays
47.	CPA3	1359	Microarray	1123	IL1R1	3554	HITS-CLIP
48.	CREB1	1385	Luciferase reporter assay//Microarray//qRT-PCR//Western blot	1124	IL1R1	3554	Biotin-Microarrays
49.	CS	1431	PAR-CLIP	1125	HDLBP	3069	CLEAR-CLIP
50.	CSRP1	1465	Microarray	1126	ID2	3398	Biotin-Microarrays
51.	CTPS1	1503	Microarray	1127	PLEKHB2	55041	Biotin-Microarrays, Microarrays
52.	CYP3A5	1577	PAR-CLIP	1128	PRKD3	23683	Biotin-Microarrays
53.	CYP7A1	1581	Luciferase reporter assay//qRT-PCR	1129	SRSF7	6432	Biotin-Microarrays
54.	DBT	1629	PAR-CLIP	1130	MSH6	2956	Biotin-Microarrays
55.	DMXL1	1657	Microarray	1131	FARSB	10056	Biotin-Microarrays
56.	DYNC1H1	1778	Microarray	1132	ALMS1	7840	Biotin-Microarrays
57.	DUSP2	1844	Luciferase reporter assay//qRT-PCR	1133	CACYBP	27101	Microarrays
58.	ECE1	1889	PAR-CLIP	1134	RALGPS2	55103	Biotin-Microarrays
59.	EGFR	1956	Luciferase reporter assay//qRT-PCR//Western blot	1135	RPL22	6146	Biotin-Microarrays
60.	EYA4	2070	Microarray	1136	SRSF4	6429	Biotin-Microarrays

61.	F2	2147	HITS-CLIP	1137	ASH1L	55870	Biotin-Microarrays
62.	F2RL1	2150	HITS-CLIP	1138	SFPQ	6421	Biotin-Microarrays
63.	FANCC	2176	HITS-CLIP	1139	RHOU	58480	Biotin-Microarrays
64.	FHL2	2274	PAR-CLIP	1140	GON4L	54856	Biotin-Microarrays
65.	FUT8	2530	Luciferase reporter assay//qRT-PCR//Western blot	1141	PRG4	10216	Biotin-Microarrays, Microarrays
66.	G6PC	2538	PAR-CLIP	1142	SRSF11	9295	Biotin-Microarrays
67.	GALNT3	2591	PAR-CLIP	1143	APH1A	51107	Microarrays
68.	GFPT1	2673	HITS-CLIP	1144	NSUN4	387338	Biotin-Microarrays
69.	B4GALT1	2683	Microarray	1145	TMED5	50999	Biotin-Microarrays
70.	GLUL	2752	PAR-CLIP	1146	PRRC2C	23215	Biotin-Microarrays
71.	GP2	2813	HITS-CLIP//PAR-CLIP	1147	VAMP4	8674	CLEAR-CLIP
72.	FFAR1	2864	PAR-CLIP	1148	RSRP1	57035	Biotin-Microarrays, Microarrays
73.	GRSF1	2926	PAR-CLIP	1149	KMT2A	4297	Biotin-Microarrays
74.	GSTM3	2947	Proteomics	1150	DDX59	83479	Biotin-Microarrays
75.	GTF2B	2959	qRT-PCR	1151	ATF6	22926	Biotin-Microarrays
76.	GTF2F1	2962	PAR-CLIP	1152	B4GALT6	9331	Biotin-Microarrays
77.	GTF2H2	2966	Microarray	1153	MYB	4602	Biotin-Microarrays, Microarrays
78.	GYS1	2997	Microarray//Northern blot//qRT-PCR//Western blot	1154	ALDH8A1	64577	Biotin-Microarrays
79.	H1F0	3005	PAR-CLIP	1155	SLC16A7	9194	Biotin-Microarrays
80.	HCCS	3052	Proteomics	1156	ZNF430	80264	Biotin-Microarrays
81.	UBE2K	3093	Proteomics	1157	TGIF2	60436	Biotin-Microarrays
82.	HLA-DQA1	3117	Microarray	1158	MFSD1	64747	Biotin-Microarrays
83.	HLA-E	3133	HITS-CLIP	1159	RAB3GAP2	25782	Biotin-Microarrays
84.	HMOX1	3162	Microarray//qRT-PCR//Western blot	1160	UBN1	29855	Biotin-Microarrays
85.	DNAJB1	3337	Microarray	1161	SATB2	23314	Biotin-Microarrays
86.	IDS	3423	HITS-CLIP//Microarray	1162	TJP2	9414	Biotin-Microarrays
87.	IFNA1	3439	Microarray	1163	CNTRL	11064	Biotin-Microarrays
88.	IFNAR2	3455	HITS-CLIP	1164	PPP6C	5537	Biotin-Microarrays
89.	IGF1R	3480	Luciferase reporter assay//qRT-	1165	HSDL2	84263	Biotin-Microarrays

			PCR//Reporter assay//Western blot				
90.	IL1A	3552	Luciferase reporter assay//qRT-PCR//Western blot	1166	DENND1A	57706	Biotin-Microarrays, Microarrays
91.	IL2RA	3559	HITS-CLIP	1167	KDSR	2531	Biotin-Microarrays
92.	FOXK2	3607	Microarray//PAR-CLIP	1168	ONECUT2	9480	Biotin-Microarrays
93.	KCNA7	3743	HITS-CLIP	1169	YLPM1	56252	Biotin-Microarrays
94.	KRT10	3858	Proteomics	1170	PGF	5228	Biotin-Microarrays
95.	KRT14	3861	Microarray	1171	RBM25	58517	Biotin-Microarrays
96.	KRT18	3875	Proteomics	1172	KLHL29	114818	Biotin-Microarrays
97.	LAMP1	3916	Microarray	1173	YPEL5	51646	Microarrays
98.	ABLIM1	3983	Microarray	1174	FAM98A	25940	Biotin-Microarrays
99.	LIMS1	3987	HITS-CLIP	1175	OGFRL1	79627	PAR-CLIP
100.	LRP3	4037	PAR-CLIP	1176	IFIT3	3437	Microarrays
101.	LYN	4067	PAR-CLIP	1177	IFIT2	3433	Microarrays
102.	MARCKS	4082	Microarray	1178	WDR11	55717	Biotin-Microarrays
103.	MAFG	4097	PAR-CLIP	1179	KANSL1	284058	Biotin-Microarrays
104.	MAZ	4150	Microarray	1180	HOXB3	3213	Biotin-Microarrays
105.	MCAM	4162	PAR-CLIP	1181	GRIA2	2891	Biotin-Microarrays
106.	MDM4	4194	PAR-CLIP	1182	KIAA1217	56243	Biotin-Microarrays
107.	MECP2	4204	Luciferase reporter assay//qRT-PCR	1183	TNFSF11	8600	Biotin-Microarrays
108.	MEF2D	4209	Luciferase reporter assay	1184	MTRF1	9617	Biotin-Microarrays
109.	MGAT1	4245	PAR-CLIP	1185	PROSER1	80209	Microarrays
110.	MKLN1	4289	HITS-CLIP	1186	KBTBD7	84078	Biotin-Microarrays
111.	MPV17	4358	Microarray	1187	NR2C1	7181	Biotin-Microarrays
112.	MSN	4478	PAR-CLIP	1188	GLT8D2	83468	Microarrays
113.	MTAP	4507	HITS-CLIP	1189	DUSP4	1846	Biotin-Microarrays
114.	MYH11	4629	HITS-CLIP	1190	TNFRSF10B	8795	HITS-CLIP
115.	NARS	4677	HITS-CLIP	1191	TNFRSF10B	8795	Microarrays
116.	NASP	4678	HITS-CLIP	1192	AKAP1	8165	Biotin-Microarrays
117.	NCAM1	4684	Luciferase reporter assay//Microarray//qRT-PCR	1193	PILRB	29990	Biotin-Microarrays
118.	NFE2L1	4779	HITS-CLIP//PAR-CLIP	1194	ZMYM2	7750	Biotin-Microarrays
119.	NFX1	4799	HITS-CLIP	1195	CXCR4	7852	Biotin-Microarrays

120.	NODAL	4838	Microarray	1196	OCRL	4952	Microarrays
121.	SLC11A2	4891	Microarray	1197	RBBP6	5930	Biotin-Microarrays
122.	OMD	4958	HITS-CLIP	1198	RPL5	6125	Biotin-Microarrays
123.	OLR1	4973	PAR-CLIP	1199	ODF2L	57489	Biotin-Microarrays
124.	ORC2	4999	Microarray	1200	CBX3	11335	Biotin-Microarrays
125.	P4HA1	5033	Luciferase reporter assay	1201	KIAA1549	57670	Biotin-Microarrays
126.	PAK1	5058	Microarray	1202	NUDT10	170685	Biotin-Microarrays
127.	PDK4	5166	Luciferase reporter assay//qRT-PCR//Western blot	1203	SLC25A16	8034	Biotin-Microarrays
128.	PFDN1	5201	Microarray	1204	MED13L	23389	PAR-CLIP, Biotin-Microarrays
129.	PFKFB2	5208	Microarray	1205	CCDC91	55297	Biotin-Microarrays
130.	PHKA1	5255	Microarray	1206	NLN	57486	Biotin-Microarrays
131.	PIK3R2	5296	PAR-CLIP	1207	ARHGAP9	64333	Microarrays
132.	PIP4K2A	5305	Microarray	1208	HOXC13	3229	Microarrays
133.	PKM	5315	Luciferase reporter assay//Microarray//PAR-CLIP//qRT-PCR//Western blot	1209	IKZF4	64375	Biotin-Microarrays
134.	PKNOX1	5316	PAR-CLIP	1210	MORF4L2	9643	Biotin-Microarrays
135.	PLAGL2	5326	Microarray	1211	KCNJ2	3759	Biotin-Microarrays
136.	PMP22	5376	Microarray	1212	RAP2C	57826	Biotin-Microarrays
137.	POLR2D	5433	HITS-CLIP	1213	GCNT7	140687	Biotin-Microarrays
138.	PPIC	5480	PAR-CLIP	1214	VAPB	9217	Biotin-Microarrays
139.	PRKAB1	5564	Luciferase reporter assay	1215	PLCG1	5335	Biotin-Microarrays
140.	MAPK1	5594	HITS-CLIP	1216	SRSF6	6431	Microarrays
141.	MAPK11	5600	Luciferase reporter assay//qRT-PCR	1217	ZNFX1	57169	Microarrays
142.	PSMB5	5693	HITS-CLIP	1218	TMEM189-UBE2V1	387522	PAR-CLIP
143.	PSMD10	5716	Proteomics	1219	SERPINB6	5269	Biotin-Microarrays
144.	PSPH	5723	Proteomics	1220	ABCC10	89845	Biotin-Microarrays
145.	QSOX1	5768	PAR-CLIP	1221	RPS10	6204	Biotin-Microarrays
146.	PTPN1	5770	Luciferase reporter assay	1222	SOX4	6659	Biotin-Microarrays
147.	PTPN2	5771	HITS-CLIP	1223	RREB1	6239	Biotin-Microarrays
148.	MAP4K2	5871	HITS-CLIP	1224	ATXN1	6310	Microarrays
149.	RAC1	5879	//Luciferase reporter	1225	NUP153	9972	Biotin-Microarrays

			assay//Microarray//qRT-PCR//Western blot				
150.	RAD21	5885	PAR-CLIP	1226	TMTC4	84899	Biotin-Microarrays
151.	RBBP5	5929	Microarray	1227	GGA3	23163	Biotin-Microarrays
152.	RBL1	5933	HITS-CLIP	1228	NUP85	79902	PAR-CLIP
153.	REL	5966	PAR-CLIP	1229	SLC25A19	60386	Microarrays
154.	RFC2	5982	HITS-CLIP	1230	PPDPF	79144	Biotin-Microarrays
155.	RPS15A	6210	HITS-CLIP	1231	POLR1B	84172	Biotin-Microarrays
156.	CLEC11A	6320	Microarray	1232	THOC2	57187	Biotin-Microarrays
157.	SCN4B	6330	Microarray	1233	RPL23	9349	Biotin-Microarrays
158.	SET	6418	Proteomics	1234	FOSB	2354	HITS-CLIP
159.	SH3GL1	6455	PAR-CLIP	1235	EML2	24139	Biotin-Microarrays
160.	ST3GAL1	6482	HITS-CLIP	1236	SYMPK	8189	Biotin-Microarrays
161.	SLC1A5	6510	HITS-CLIP	1237	AP5S1	55317	CLEAR-CLIP
162.	SLC2A3	6515	PAR-CLIP	1238	RRBP1	6238	Biotin-Microarrays
163.	SLC4A1	6521	PAR-CLIP	1239	ZNF133	7692	Biotin-Microarrays
164.	SLC7A1	6541	Luciferase reporter assay//Northern blot//qRT-PCR//Reporter assay//Western blot	1240	FLRT3	23767	Biotin-Microarrays
165.	SLC9A1	6548	Microarray//PAR-CLIP	1241	TBC1D20	128637	Biotin-Microarrays
166.	SLC15A2	6565	Microarray	1242	ST3GAL3	6487	PAR-CLIP
167.	SNTB2	6645	HITS-CLIP	1243	CAPNS1	826	HITS-CLIP
168.	SOX2	6657	Microarray	1244	THRA	7067	Biotin-Microarrays
169.	SPIB	6689	HITS-CLIP	1245	KTN1	3895	Biotin-Microarrays
170.	SRF	6722	Luciferase reporter assay//qRT-PCR//Western blot	1246	TIMM8A	1678	Biotin-Microarrays
171.	SSR3	6747	Microarray	1247	CANX	821	Biotin-Microarrays, CLEAR-CLIP
172.	SSTR2	6752	HITS-CLIP	1248	IPPK	64768	Biotin-Microarrays
173.	STXBP2	6813	HITS-CLIP	1249	EMC1	23065	Biotin-Microarrays
174.	ADAM17	6868	Luciferase reporter assay//qRT-PCR	1250	PLA2G5	5322	Biotin-Microarrays
175.	TCP11	6954	Microarray	1251	UBR4	23352	HITS-CLIP
176.	TEP1	7011	HITS-CLIP	1252	CHTF18	63922	Biotin-Microarrays

177.	TERF2	7014	HITS-CLIP	1253	GNG13	51764	Microarrays
178.	TFDP2	7029	PAR-CLIP	1254	MACF1	23499	Biotin-Microarrays
179.	TGFB1	7040	qRT-PCR	1255	SMARCA4	6597	Biotin-Microarrays
180.	TIAL1	7073	HITS-CLIP	1256	KDM4B	23030	Biotin-Microarrays
181.	TPD52L2	7165	Luciferase reporter assay//Microarray//qRT-PCR	1257	PTPN12	5782	Biotin-Microarrays
182.	UBE2G2	7327	HITS-CLIP	1258	PEX1	5189	Biotin-Microarrays
183.	UBE2L3	7332	Microarray	1259	LRFN1	57622	Biotin-Microarrays
184.	SUMO1	7341	PAR-CLIP	1260	SRD5A3	79644	Microarrays
185.	UBTF	7343	PAR-CLIP	1261	GNAZ	2781	Biotin-Microarrays
186.	UROS	7390	PAR-CLIP	1262	KRT17	3872	Microarrays
187.	VDR	7421	PAR-CLIP	1263	FOXP2	93986	PAR-CLIP, Biotin-Microarrays
188.	VEGFC	7424	ELISA//Luciferase reporter assay//qRT-PCR//Western blot	1264	STRIP2	57464	Microarrays
189.	VHL	7428	Microarray	1265	DNAJB9	4189	Microarrays
190.	WNT1	7471	Luciferase reporter assay//qRT-PCR//Western blot	1266	NDUFA5	4698	Biotin-Microarrays
191.	YWHAB	7529	HITS-CLIP//PAR-CLIP	1267	GAD1	2571	Biotin-Microarrays
192.	ZNF74	7625	HITS-CLIP	1268	SNRPN	6638	Biotin-Microarrays
193.	ZNF154	7710	PAR-CLIP	1269	MYO5C	55930	Microarrays
194.	ZNF226	7769	PAR-CLIP	1270	TTBK2	146057	Biotin-Microarrays
195.	LUZP1	7798	Microarray	1271	VPS13C	54832	Biotin-Microarrays
196.	MAFK	7975	PAR-CLIP	1272	PALLD	23022	Biotin-Microarrays
197.	ARHGEF5	7984	HITS-CLIP	1273	RPAIN	84268	Biotin-Microarrays
198.	MLF2	8079	PAR-CLIP	1274	AIPL1	23746	Biotin-Microarrays
199.	SLC7A5	8140	PAR-CLIP	1275	PHF20L1	51105	Biotin-Microarrays
200.	SLC10A3	8273	PAR-CLIP	1276	ILF3	3609	Biotin-Microarrays
201.	IFITM1	8519	PAR-CLIP	1277	MTUS1	57509	Biotin-Microarrays
202.	DGKE	8526	HITS-CLIP	1278	MIS18BP1	55320	Biotin-Microarrays
203.	MAPKAPK5	8550	PAR-CLIP	1279	SEC14L1	6397	Biotin-Microarrays
204.	PRKRA	8575	Luciferase reporter assay//Microarray//qRT-PCR//Western blot	1280	RHBDF2	79651	Biotin-Microarrays
205.	SOCS1	8651	GFP reporter assay	1281	PHF10	55274	Biotin-Microarrays

206.	PEA15	8682	Microarray	1282	SAT1	6303	Microarrays
207.	TNFSF14	8740	PAR-CLIP	1283	PXDN	7837	Biotin-Microarrays
208.	SUCLA2	8803	Microarray	1284	GDF15	9518	Microarrays
209.	CDKL1	8814	PAR-CLIP	1285	JUND	3727	Biotin-Microarrays
210.	WASF1	8936	Microarray	1286	CAMSAP1	157922	Biotin-Microarrays
211.	SPAG9	9043	Microarray	1287	H19	283120	Biotin-Microarrays
212.	TBX19	9095	Luciferase reporter assay//qRT-PCR	1288	COL5A1	1289	Biotin-Microarrays
213.	USP10	9100	Microarray	1289	LAMA5	3911	Biotin-Microarrays
214.	SLC16A5	9121	HITS-CLIP	1290	UCK1	83549	Biotin-Microarrays
215.	LRAT	9227	HITS-CLIP	1291	YIPF2	78992	Biotin-Microarrays
216.	RPS6KA5	9252	HITS-CLIP	1292	EIF2S3	1968	Biotin-Microarrays
217.	NUMBL	9253	Luciferase reporter assay//qRT-PCR	1293	SESN2	83667	Biotin-Microarrays
218.	CD83	9308	Microarray	1294	SLC35D2	11046	Biotin-Microarrays
219.	EFTUD2	9343	PAR-CLIP	1295	RBM39	9584	Biotin-Microarrays
220.	TGFBRAP1	9392	Microarray	1296	GSE1	23199	Biotin-Microarrays
221.	ZNF264	9422	Microarray	1297	RLIM	51132	Biotin-Microarrays
222.	MED7	9443	HITS-CLIP	1298	ANKHD1	54882	Biotin-Microarrays
223.	CHST3	9469	Microarray	1299	DIAPH1	1729	Biotin-Microarrays
224.	AKAP5	9495	PAR-CLIP	1300	MAP1B	4131	Biotin-Microarrays
225.	NPEPPS	9520	Microarray	1301	TOP2A	7153	Biotin-Microarrays
226.	ENTPD4	9583	Luciferase reporter assay//qRT-PCR	1302	STARD3	10948	Microarrays
227.	PHF14	9678	Microarray	1303	PRKAB2	5565	Biotin-Microarrays
228.	TRAM2	9697	Microarray	1304	ZNF304	57343	Biotin-Microarrays
229.	KIAA0100	9703	PAR-CLIP	1305	SNRPA1	6627	Biotin-Microarrays
230.	PCLAF	9768	Microarray	1306	C19orf12	83636	Microarrays
231.	BMS1	9790	HITS-CLIP	1307	RAF1	5894	Biotin-Microarrays
232.	IST1	9798	PAR-CLIP	1308	NUP210	23225	Biotin-Microarrays
233.	TOMM70	9868	PAR-CLIP	1309	ENOSF1	55556	Biotin-Microarrays
234.	FIG4	9896	HITS-CLIP	1310	IMMT	10989	Biotin-Microarrays
235.	NCAPD2	9918	PAR-CLIP	1311	IQCA1	79781	Biotin-Microarrays
236.	HS3ST1	9957	HITS-CLIP	1312	PRKAA1	5562	Biotin-Microarrays

237.	CCS	9973	HITS-CLIP	1313	PNISR	25957	Biotin-Microarrays
238.	AKT3	10000	Immunoblot//Luciferase reporter assay//qRT-PCR	1314	POPDC3	64208	Biotin-Microarrays
239.	ABCF2	10061	HITS-CLIP//PAR-CLIP	1315	KDM6B	23135	Biotin-Microarrays
240.	OPTN	10133	HITS-CLIP	1316	VPS13B	157680	Biotin-Microarrays
241.	PRMT3	10196	PAR-CLIP	1317	SNAP25	6616	Biotin-Microarrays
242.	TRIB1	10221	Luciferase reporter assay//qRT-PCR	1318	DCAF8	50717	Biotin-Microarrays
243.	DCAF7	10238	HITS-CLIP	1319	ZMYM5	9205	Biotin-Microarrays
244.	CALCOCO2	10241	HITS-CLIP	1320	DCLK1	9201	Biotin-Microarrays
245.	SPRY2	10253	Luciferase reporter assay//Microarray//qRT-PCR//Western blot	1321	RFXAP	5994	Biotin-Microarrays
246.	FSTL3	10272	Microarray	1322	GPALPP1	55425	Biotin-Microarrays
247.	PRSS16	10279	PAR-CLIP	1323	WDR74	54663	PAR-CLIP
248.	SF3A1	10291	HITS-CLIP	1324	SPINK5	11005	Microarrays
249.	SEC23B	10483	HITS-CLIP	1325	COX16	51241	Biotin-Microarrays
250.	SPTLC1	10558	Microarray	1326	MED6	10001	Biotin-Microarrays
251.	SLC19A2	10560	Microarray	1327	MRPS36	92259	Biotin-Microarrays
252.	CDC42EP3	10602	Microarray	1328	IRAK2	3656	CLEAR-CLIP
253.	YKT6	10652	Microarray	1329	PRPF38B	55119	Biotin-Microarrays
254.	GMEB1	10691	HITS-CLIP	1330	VAV3	10451	Biotin-Microarrays
255.	YME1L1	10730	HITS-CLIP	1331	SORT1	6272	Luciferase Reporter Assay
256.	KIF1C	10749	HITS-CLIP	1332	PPHLN1	51535	Biotin-Microarrays
257.	TOB2	10766	Microarray//PAR-CLIP	1333	FKBP11	51303	Biotin-Microarrays
258.	RAB10	10890	HITS-CLIP	1334	TMEM106C	79022	Biotin-Microarrays
259.	GPR75	10936	PAR-CLIP	1335	IAH1	285148	Biotin-Microarrays
260.	KDELR1	10945	HITS-CLIP	1336	NAV1	89796	Biotin-Microarrays
261.	AP3M2	10947	Luciferase reporter assay//qRT-PCR	1337	TMEM241	85019	Biotin-Microarrays
262.	PNRC1	10957	HITS-CLIP	1338	TPGS2	25941	Biotin-Microarrays
263.	ERP29	10961	Proteomics	1339	COL4A2	1284	Biotin-Microarrays
264.	LILRA2	11027	HITS-CLIP	1340	ARGLU1	55082	Biotin-Microarrays

265.	BTN3A2	11118	HITS-CLIP	1341	BIVM	54841	Biotin-Microarrays
266.	KAT7	11143	HITS-CLIP	1342	TBX3	6926	Biotin-Microarrays
267.	NUDT3	11165	PAR-CLIP	1343	OASL	8638	Microarrays
268.	AKAP11	11215	Microarray	1344	HRK	8739	Biotin-Microarrays
269.	GALNT6	11226	PAR-CLIP	1345	CEP162	22832	Biotin-Microarrays
270.	PXMP4	11264	HITS-CLIP	1346	EHF	26298	Biotin-Microarrays
271.	Cux1	13047	qRT-PCR	1347	CAPRIN1	4076	PAR-CLIP
272.	HSPA4L	22824	PAR-CLIP	1348	AVIL	10677	Biotin-Microarrays
273.	SLITRK3	22865	HITS-CLIP	1349	KRT7	3855	Microarrays
274.	MLXIP	22877	PAR-CLIP	1350	HNRNPA1	3178	Biotin-Microarrays
275.	FOXJ3	22887	Luciferase reporter assay//qRT-PCR	1351	AHI1	54806	Biotin-Microarrays
276.	MAPRE1	22919	Microarray	1352	USP15	9958	Microarrays
277.	POMZP3	22932	Microarray	1353	DYNC1LI2	1783	Biotin-Microarrays
278.	FBXO21	23014	PAR-CLIP	1354	TAF5L	27097	Microarrays
279.	UNC13A	23025	HITS-CLIP	1355	STX6	10228	Luciferase Reporter Assay
280.	SMG1	23049	HITS-CLIP	1356	DHX9	1660	Biotin-Microarrays
281.	NMNAT2	23057	Microarray	1357	CEP350	9857	Biotin-Microarrays
282.	ERC1	23085	PAR-CLIP	1358	LAMC1	3915	Microarrays
283.	PEG10	23089	Luciferase reporter assay	1359	COX5B	1329	Biotin-Microarrays
284.	CDK19	23097	PAR-CLIP	1360	REV1	51455	Biotin-Microarrays
285.	NCDN	23154	Microarray	1361	ARHGEF4	50649	Biotin-Microarrays
286.	MPRIP	23164	PAR-CLIP	1362	FLNB	2317	Biotin-Microarrays
287.	TTLL12	23170	HITS-CLIP	1363	PHF11	51131	Biotin-Microarrays
288.	LPIN1	23175	Luciferase reporter assay//qRT-PCR//Western blot	1364	LMO7	4008	Biotin-Microarrays
289.	XPO6	23214	Luciferase reporter assay//qRT-PCR	1365	SPDYE1	285955	Biotin-Microarrays
290.	SIK2	23235	HITS-CLIP	1366	IGF2BP3	10643	Biotin-Microarrays
291.	WWC1	23286	HITS-CLIP	1367	IL6	3569	Microarrays
292.	CTDNEP1	23399	Luciferase reporter assay//qRT-PCR//Western blot	1368	SRSF1	6426	Biotin-Microarrays
293.	GPR161	23432	PAR-CLIP	1369	DNAJC1	64215	Biotin-Microarrays
294.	SLC44A1	23446	Microarray	1370	CDK5RAP2	55755	Biotin-Microarrays
295.	ABCA6	23460	HITS-CLIP	1371	GARNL3	84253	Biotin-Microarrays

296.	QPRT	23475	HITS-CLIP	1372	PSMB7	5695	Biotin-Microarrays
297.	POFUT1	23509	Microarray	1373	MYC	4609	qPCR, Western Blot
298.	ZNF281	23528	HITS-CLIP	1374	DENND4C	55667	Biotin-Microarrays
299.	DNPEP	23549	HITS-CLIP	1375	KIF13A	63971	Biotin-Microarrays
300.	HEBP2	23593	HITS-CLIP	1376	YIPF3	25844	PAR-CLIP
301.	ORC6	23594	PAR-CLIP	1377	FOXF2	2295	Microarrays
302.	ACOT9	23597	HITS-CLIP	1378	IER3	8870	Biotin-Microarrays
303.	PATZ1	23598	PAR-CLIP	1379	NUMA1	4926	PAR-CLIP
304.	PHLDA3	23612	HITS-CLIP	1380	CCDC90B	60492	Biotin-Microarrays
305.	TRIM29	23650	Microarray	1381	TGS1	96764	Biotin-Microarrays
306.	SLC7A11	23657	Luciferase reporter assay//qRT-PCR	1382	NEK1	4750	Biotin-Microarrays
307.	OSBP2	23762	Microarray	1383	KIF23	9493	Biotin-Microarrays
308.	MAFF	23764	HITS-CLIP	1384	HAUS2	55142	Biotin-Microarrays
309.	DSTYK	25778	Luciferase reporter assay//qRT-PCR	1385	UACA	55075	Biotin-Microarrays
310.	FBXO7	25793	Microarray	1386	IFI44	10561	Microarrays
311.	BAMBI	25805	HITS-CLIP	1387	CYP1B1	1545	Biotin-Microarrays
312.	MTO1	25821	HITS-CLIP	1388	RAB1A	5861	Biotin-Microarrays
313.	TECPR1	25851	HITS-CLIP	1389	PREPL	9581	Biotin-Microarrays
314.	CLIC4	25932	Luciferase reporter assay//Microarray//PAR-CLIP//qRT-PCR	1390	FBXO11	80204	Biotin-Microarrays
315.	HEATR5A	25938	PAR-CLIP	1391	ATRAID	51374	Microarrays
316.	RAI14	26064	Microarray	1392	LRPPRC	10128	Biotin-Microarrays
317.	PYGO1	26108	Microarray	1393	MYOF	26509	Biotin-Microarrays
318.	WSB1	26118	PAR-CLIP	1394	KIF11	3832	Biotin-Microarrays, Microarrays
319.	ZNF658	26149	Microarray	1395	CEP55	55165	Biotin-Microarrays, Microarrays
320.	MYCBP	26292	HITS-CLIP//Microarray	1396	GPR87	53836	Microarrays
321.	STAU2	27067	Microarray	1397	ATIC	471	HITS-CLIP
322.	FOXP1	27086	Luciferase reporter assay//qRT-PCR	1398	IDH1	3417	Biotin-Microarrays
323.	ST6GALN	27090	Microarray	1399	SENP7	57337	Biotin-Microarrays

	AC4						
324.	TNRC6A	27327	PAR-CLIP	1400	FAM13A	10144	Biotin-Microarrays
325.	RABGEF1	27342	Microarray	1401	AP1AR	55435	Biotin-Microarrays
326.	PHPT1	29085	Proteomics	1402	HNRNPD	3184	Biotin-Microarrays
327.	RACGAP1	29127	PAR-CLIP	1403	KIAA1109	84162	Biotin-Microarrays
328.	PARVB	29780	HITS-CLIP//PAR-CLIP	1404	BMPR1B	658	Biotin-Microarrays
329.	OLA1	29789	HITS-CLIP	1405	LARP1B	55132	Biotin-Microarrays
330.	GPSM2	29899	PAR-CLIP	1406	PRDM5	11107	Biotin-Microarrays
331.	EEF2K	29904	PAR-CLIP	1407	TRPC3	7222	Biotin-Microarrays
332.	AK3	50808	PAR-CLIP	1408	NUP54	53371	Biotin-Microarrays
333.	HDDC2	51020	PAR-CLIP	1409	CXCL9	4283	Biotin-Microarrays
334.	GLOD4	51031	Proteomics	1410	PPA2	27068	Biotin-Microarrays
335.	TUBD1	51174	PAR-CLIP	1411	FBN2	2201	Biotin-Microarrays
336.	ACP6	51205	HITS-CLIP	1412	ETV6	2120	Biotin-Microarrays
337.	NT5C3A	51251	Luciferase reporter assay	1413	C1RL	51279	Biotin-Microarrays
338.	NIP7	51388	HITS-CLIP	1414	SCAF11	9169	Biotin-Microarrays
339.	CINP	51550	HITS-CLIP	1415	TMTC3	160418	Biotin-Microarrays
340.	RAB6B	51560	Luciferase reporter assay//qRT-PCR	1416	KERA	11081	Microarrays
341.	GDE1	51573	HITS-CLIP	1417	SYCP3	50511	Biotin-Microarrays
342.	ESF1	51575	HITS-CLIP	1418	GAS2L3	283431	Biotin-Microarrays
343.	MRPS23	51649	PAR-CLIP	1419	TDG	6996	Microarrays
344.	MSRB1	51734	PAR-CLIP	1420	SMARCC2	6601	Biotin-Microarrays
345.	NLGN3	54413	Microarray	1421	CSAD	51380	Biotin-Microarrays
346.	TAS2R5	54429	HITS-CLIP//PAR-CLIP	1422	LMBR1L	55716	Biotin-Microarrays
347.	RNF216	54476	HITS-CLIP	1423	TMBIM6	7009	Microarrays
348.	TMCO1	54499	HITS-CLIP	1424	RBM26	64062	Biotin-Microarrays
349.	RBM47	54502	PAR-CLIP	1425	MBNL2	10150	Biotin-Microarrays
350.	GNL3L	54552	Microarray	1426	CUL4A	8451	Biotin-Microarrays
351.	INO80	54617	HITS-CLIP	1427	SLC12A6	9990	Biotin-Microarrays
352.	MED18	54797	HITS-CLIP	1428	TCF12	6938	Biotin-Microarrays
353.	PIGG	54872	HITS-CLIP	1429	ZSCAN29	146050	Biotin-Microarrays
354.	ZCCHC2	54877	Microarray	1430	GTF2A2	2958	Biotin-Microarrays
355.	RHBDL2	54933	HITS-CLIP	1431	BCL2A1	597	Biotin-Microarrays

356.	PIGX	54965	HITS-CLIP	1432	WDR61	80349	Biotin-Microarrays
357.	FAM118A	55007	Microarray	1433	PCSK6	5046	Biotin-Microarrays
358.	PTCD3	55037	HITS-CLIP	1434	CYP1A2	1544	Biotin-Microarrays
359.	RBM23	55147	HITS-CLIP	1435	ABHD2	11057	Biotin-Microarrays
360.	SBNO1	55206	HITS-CLIP	1436	MFGE8	4240	Biotin-Microarrays
361.	VPS53	55275	HITS-CLIP	1437	UNC45A	55898	Biotin-Microarrays
362.	C4orf19	55286	PAR-CLIP	1438	MCTP2	55784	Biotin-Microarrays
363.	TMEM40	55287	HITS-CLIP	1439	FURIN	5045	Microarrays
364.	SPTLC3	55304	HITS-CLIP	1440	IQGAP1	8826	Biotin-Microarrays, Microarrays
365.	CHDH	55349	HITS-CLIP	1441	ZFHX3	463	Biotin-Microarrays
366.	NCBP3	55421	HITS-CLIP	1442	NLRC5	84166	Biotin-Microarrays
367.	CHST12	55501	Microarray	1443	PDPK1	5170	Biotin-Microarrays
368.	SLC35E3	55508	HITS-CLIP	1444	TCF25	22980	HITS-CLIP, Biotin-Microarrays
369.	RBM38	55544	PAR-CLIP	1445	ESCO1	114799	Biotin-Microarrays
370.	GALNT10	55568	Immunohistochemistry//Immunoprecipitaion//In situ hybridization//Luciferase reporter assay//qRT-PCR//Western blot	1446	GREB1L	80000	Biotin-Microarrays
371.	NAGK	55577	HITS-CLIP	1447	PELP1	27043	Microarrays
372.	MED29	55588	HITS-CLIP	1448	MINK1	50488	Biotin-Microarrays
373.	ANKRD10	55608	Microarray	1449	TP53	7157	Biotin-Microarrays
374.	TBC1D22B	55633	Microarray	1450	TMC6	11322	Biotin-Microarrays
375.	MREG	55686	PAR-CLIP	1451	SLC16A3	9123	Biotin-Microarrays
376.	LUC7L	55692	HITS-CLIP	1452	SECTM1	6398	Biotin-Microarrays
377.	HHAT	55733	Microarray	1453	RNF165	494470	Biotin-Microarrays
378.	PRR11	55771	Microarray	1454	SMAD4	4089	Biotin-Microarrays
379.	METTL2B	55798	HITS-CLIP	1455	NFIC	4782	Biotin-Microarrays
380.	LRP2BP	55805	HITS-CLIP	1456	PRDM15	63977	Biotin-Microarrays
381.	EMC3	55831	PAR-CLIP	1457	AKT1	207	qPCR, Western Blot
382.	UBAP2	55833	Luciferase reporter assay//qRT-PCR	1458	EVI5L	115704	HITS-CLIP
383.	ZC3H15	55854	HITS-CLIP	1459	RERE	473	Microarrays

384.	ZNF395	55893	GFP reporter assay	1460	EPHA2	1969	HITS-CLIP
385.	KLHL7	55975	PAR-CLIP	1461	LMO4	8543	Biotin-Microarrays
386.	ALG1	56052	HITS-CLIP	1462	ATP1B1	481	Biotin-Microarrays
387.	TDRD1	56165	HITS-CLIP	1463	DCAF6	55827	Biotin-Microarrays
388.	SAR1A	56681	HITS-CLIP	1464	POU2F1	5451	Biotin-Microarrays
389.	KCMF1	56888	HITS-CLIP	1465	XPR1	9213	Microarrays
390.	PARP11	57097	Microarray	1466	TOR1AIP1	26092	Biotin-Microarrays
391.	LYRM4	57128	HITS-CLIP	1467	ADAMTSL4	54507	Biotin-Microarrays
392.	APMAP	57136	Proteomics	1468	PI4KB	5298	PAR-CLIP
393.	JPH2	57158	HITS-CLIP	1469	ENSA	2029	Biotin-Microarrays, Microarrays
394.	CLK4	57396	HITS-CLIP	1470	GOLPH3L	55204	Microarrays
395.	CYP20A1	57404	HITS-CLIP	1471	INTS7	25896	Biotin-Microarrays
396.	KIAA1143	57456	HITS-CLIP	1472	KCNN3	3782	PAR-CLIP
397.	ESYT2	57488	HITS-CLIP	1473	GATAD2B	57459	Biotin-Microarrays
398.	XPO5	57510	HITS-CLIP	1474	ARF1	375	Biotin-Microarrays
399.	HECW2	57520	Microarray	1475	CDC42BPA	8476	Biotin-Microarrays
400.	PDP2	57546	HITS-CLIP	1476	PLEKHA6	22874	HITS-CLIP, Biotin-Microarrays
401.	EP400	57634	Microarray	1477	ASXL2	55252	Biotin-Microarrays
402.	USP28	57646	Microarray	1478	SNRNP200	23020	Biotin-Microarrays
403.	ZBTB4	57659	Microarray	1479	MRPS5	64969	Biotin-Microarrays
404.	ZFP14	57677	HITS-CLIP	1480	DQX1	165545	Microarrays
405.	CPNE5	57699	Microarray	1481	SLC20A1	6574	Biotin-Microarrays
406.	POLD4	57804	PAR-CLIP	1482	SCRN3	79634	Biotin-Microarrays
407.	MRPL17	63875	HITS-CLIP	1483	TMEFF2	23671	Biotin-Microarrays
408.	FAM217B	63939	HITS-CLIP	1484	CDCA7	83879	Biotin-Microarrays
409.	CLSPN	63967	Microarray	1485	PHOSPHO2	493911	Biotin-Microarrays
410.	NOD2	64127	Luciferase reporter assay//qRT-PCR//Western blot	1486	CCDC150	284992	Biotin-Microarrays
411.	ZNF106	64397	PAR-CLIP	1487	RHBDD1	84236	Biotin-Microarrays, PAR-CLIP, Microarrays
412.	TMEM168	64418	HITS-CLIP	1488	ACKR3	57007	Biotin-Microarrays
413.	MRPS25	64432	HITS-CLIP	1489	COPS7B	64708	Biotin-Microarrays
414.	NOM1	64434	HITS-CLIP	1490	DIS3L2	129563	Biotin-Microarrays

415.	SMURF2	64750	Microarray	1491	TAMM41	132001	Biotin-Microarrays
416.	MEAF6	64769	HITS-CLIP	1492	VGLL4	9686	Biotin-Microarrays
417.	EFCAB6	64800	Proteomics	1493	RBMS3	27303	Biotin-Microarrays
418.	KRI1	65095	PAR-CLIP	1494	GOLGA4	2803	Biotin-Microarrays
419.	AACS	65985	Luciferase reporter assay//qRT-PCR	1495	CTDSPL	10217	Biotin-Microarrays, Microarrays
420.	FUNDC2	65991	Luciferase reporter assay//qRT-PCR	1496	TMF1	7110	Biotin-Microarrays
421.	CENPM	79019	HITS-CLIP	1497	LRIG1	26018	HITS-CLIP
422.	ZNF655	79027	HITS-CLIP	1498	NFKBIZ	64332	Biotin-Microarrays
423.	GLB1L	79411	HITS-CLIP	1499	COL8A1	1295	Biotin-Microarrays
424.	ATP13A3	79572	Microarray	1500	NCEH1	57552	Microarrays
425.	NKAP	79576	HITS-CLIP	1501	LPP	4026	Biotin-Microarrays
426.	SLC52A2	79581	Microarray	1502	TMEM44	93109	Biotin-Microarrays
427.	PGBD5	79605	HITS-CLIP	1503	SCD5	79966	Biotin-Microarrays
428.	HECTD3	79654	Microarray	1504	USP53	54532	Biotin-Microarrays
429.	ZYG11B	79699	HITS-CLIP//PAR-CLIP	1505	CBR4	84869	Biotin-Microarrays
430.	TBL1XR1	79718	Microarray	1506	MYO10	4651	Biotin-Microarrays
431.	MOB3B	79817	Microarray	1507	RPL37	6167	Biotin-Microarrays
432.	MFSD13A	79847	HITS-CLIP	1508	BTF3	689	Biotin-Microarrays
433.	SNX22	79856	HITS-CLIP	1509	FBXL17	64839	Biotin-Microarrays
434.	ACTR5	79913	HITS-CLIP	1510	DDX46	9879	Biotin-Microarrays
435.	ADM2	79924	HITS-CLIP	1511	RNF145	153830	Biotin-Microarrays
436.	TNIP3	79931	HITS-CLIP	1512	G3BP1	10146	Biotin-Microarrays
437.	SYNPO2L	79933	HITS-CLIP	1513	GFOD1	54438	PAR-CLIP
438.	SLC35E1	79939	HITS-CLIP	1514	CDKAL1	54901	Biotin-Microarrays
439.	PLEKHS1	79949	HITS-CLIP//PAR-CLIP	1515	SCUBE3	222663	Biotin-Microarrays
440.	NOL10	79954	PAR-CLIP	1516	SLC22A3	6581	Biotin-Microarrays
441.	CCDC170	80129	PAR-CLIP	1517	VWDE	221806	Biotin-Microarrays
442.	NAA50	80218	HITS-CLIP	1518	PURB	5814	HITS-CLIP
443.	RAB11FIP 1	80223	HITS-CLIP//Luciferase reporter assay//qRT-PCR	1519	ATXN7L1	222255	Biotin-Microarrays
444.	ORAI2	80228	HITS-CLIP//PAR-CLIP	1520	TRIM4	89122	Biotin-Microarrays
445.	CXorf21	80231	HITS-CLIP	1521	SPIN2A	54466	Biotin-Microarrays

446.	HM13	81502	PAR-CLIP	1522	OGT	8473	Biotin-Microarrays
447.	ANKRD13C	81573	Microarray	1523	MFHAS1	9258	Biotin-Microarrays
448.	NIPA2	81614	PAR-CLIP	1524	SLC25A37	51312	Biotin-Microarrays
449.	TIGD6	81789	HITS-CLIP	1525	DOCK5	80005	Biotin-Microarrays
450.	RNF170	81790	HITS-CLIP//Microarray	1526	ERLIN2	11160	Biotin-Microarrays
451.	THAP2	83591	HITS-CLIP	1527	SLC26A7	115111	Biotin-Microarrays
452.	KATNAL1	84056	Microarray	1528	MTDH	92140	HITS-CLIP
453.	MAGT1	84061	HITS-CLIP	1529	ZCCHC7	84186	Biotin-Microarrays
454.	MRI1	84245	HITS-CLIP	1530	ASTN2	23245	Biotin-Microarrays
455.	YIPF4	84272	HITS-CLIP//PAR-CLIP	1531	SURF1	6834	Biotin-Microarrays
456.	SLC25A33	84275	HITS-CLIP	1532	SH3GLB2	56904	Microarrays
457.	MCM8	84515	PAR-CLIP	1533	NOTCH1	4851	Biotin-Microarrays
458.	DCTN5	84516	Microarray	1534	POLR3A	11128	Biotin-Microarrays
459.	MT4	84560	Microarray	1535	HERC4	26091	Biotin-Microarrays
460.	MFSD14B	84641	PAR-CLIP	1536	PPRC1	23082	Biotin-Microarrays
461.	ZNF347	84671	PAR-CLIP	1537	ITPRIP	85450	Biotin-Microarrays
462.	PPP1R9B	84687	Microarray	1538	CNNM2	54805	Biotin-Microarrays
463.	PIGO	84720	HITS-CLIP//PAR-CLIP	1539	EIF3M	10480	Biotin-Microarrays
464.	CNDP1	84735	HITS-CLIP	1540	CELF1	10658	Biotin-Microarrays
465.	LMNB2	84823	Microarray//PAR-CLIP	1541	INTS4	92105	Biotin-Microarrays
466.	LRCH3	84859	HITS-CLIP	1542	ATM	472	Biotin-Microarrays
467.	CCDC142	84865	HITS-CLIP	1543	ST14	6768	Microarrays
468.	ADO	84890	Microarray	1544	ADAM33	80332	Biotin-Microarrays
469.	POMGNT2	84892	Microarray	1545	TMEM25	84866	PAR-CLIP
470.	PLXDC2	84898	HITS-CLIP//PAR-CLIP	1546	SOGA1	140710	Biotin-Microarrays
471.	NFATC2IP	84901	Luciferase reporter assay//qRT-PCR	1547	LSM14B	149986	Biotin-Microarrays
472.	CEP89	84902	HITS-CLIP	1548	ITGB1	3688	Biotin-Microarrays
473.	UTP4	84916	HITS-CLIP//PAR-CLIP	1549	LATS2	26524	Biotin-Microarrays
474.	LRP11	84918	Microarray	1550	GXYLT1	283464	Biotin-Microarrays
475.	MASTL	84930	HITS-CLIP	1551	DIP2C	22982	Biotin-Microarrays
476.	SERAC1	84947	Microarray	1552	NPAS3	64067	Biotin-Microarrays

477.	PRPF38A	84950	PAR-CLIP	1553	KCTD14	65987	Biotin-Microarrays
478.	RRP36	88745	HITS-CLIP	1554	ME3	10873	Biotin-Microarrays
479.	SIGLEC12	89858	PAR-CLIP	1555	C4orf33	132321	Biotin-Microarrays
480.	C16orf45	89927	HITS-CLIP	1556	PTPRO	5800	Biotin-Microarrays
481.	TMEM250	90120	HITS-CLIP	1557	VTI1A	143187	Biotin-Microarrays
482.	ZSWIM1	90204	HITS-CLIP//PAR-CLIP	1558	MMAA	166785	Biotin-Microarrays
483.	ZNF160	90338	Microarray	1559	ZNF827	152485	Biotin-Microarrays
484.	TIMM29	90580	HITS-CLIP//PAR-CLIP	1560	TMEM45B	120224	Microarrays
485.	STARD13	90627	Microarray	1561	BICD1	636	Biotin-Microarrays
486.	MFSD4B	91749	HITS-CLIP//PAR-CLIP	1562	PABPC3	5042	Biotin-Microarrays
487.	LIN52	91750	HITS-CLIP	1563	DST	667	Biotin-Microarrays
488.	SPECC1	92521	HITS-CLIP	1564	MZT2B	80097	Biotin-Microarrays
489.	G6PC3	92579	Luciferase reporter assay//Microarray//qRT-PCR	1565	EPG5	57724	Biotin-Microarrays
490.	TIMM50	92609	PAR-CLIP	1566	DCLRE1C	64421	Biotin-Microarrays
491.	SYAP1	94056	HITS-CLIP	1567	NMT2	9397	Biotin-Microarrays
492.	EGLN3	112399	Luciferase reporter assay//qRT-PCR	1568	USP12	219333	HITS-CLIP
493.	GTF3C6	112495	Microarray	1569	MBNL1	4154	Biotin-Microarrays
494.	XKR4	114786	HITS-CLIP	1570	GJA1	2697	Biotin-Microarrays
495.	RNF157	114804	HITS-CLIP	1571	FARP1	10160	CLEAR-CLIP
496.	SMYD4	114826	PAR-CLIP	1572	HNRNPDL	9987	Biotin-Microarrays
497.	OSBPL10	114884	HITS-CLIP//PAR-CLIP	1573	BCL2L11	10018	Biotin-Microarrays
498.	FLYWCH 2	114984	HITS-CLIP	1574	RANBP2	5903	Biotin-Microarrays
499.	WDR31	114987	HITS-CLIP	1575	SLC25A27	9481	Biotin-Microarrays
500.	ZNF618	114991	Microarray	1576	CMTM7	112616	PAR-CLIP
501.	BATF2	116071	Microarray	1577	CFDP1	10428	Biotin-Microarrays
502.	FAM210B	116151	PAR-CLIP	1578	CMIP	80790	Biotin-Microarrays
503.	MOGAT1	116255	HITS-CLIP	1579	TRIP12	9320	HITS-CLIP
504.	WDR17	116966	HITS-CLIP//PAR-CLIP	1580	KCTD15	79047	Biotin-Microarrays
505.	SLC16A10	117247	HITS-CLIP	1581	DDAH1	23576	Microarrays
506.	ZNF354B	117608	HITS-CLIP//PAR-CLIP	1582	MSI2	124540	Biotin-Microarrays
507.	BORCS7	119032	PAR-CLIP	1583	JPH3	57338	Biotin-Microarrays

508.	ANKRD9	122416	HITS-CLIP	1584	ANKH	56172	Biotin-Microarrays
509.	MRPL52	122704	PAR-CLIP	1585	TBRG1	84897	Biotin-Microarrays
510.	GPHB5	122876	Microarray	1586	ABCA5	23461	Biotin-Microarrays
511.	CYB5D1	124637	HITS-CLIP	1587	OBSCN	84033	Biotin-Microarrays
512.	KRBA2	124751	HITS-CLIP	1588	LONRF1	91694	Microarrays
513.	CCDC43	124808	PAR-CLIP	1589	PPP1R3A	5506	Biotin-Microarrays
514.	ZNF573	126231	HITS-CLIP	1590	SH3RF1	57630	Biotin-Microarrays
515.	FBXO27	126433	PAR-CLIP	1591	BUB3	9184	CLEAR-CLIP
516.	RNF19B	127544	HITS-CLIP	1592	FGD5	152273	Microarrays
517.	C1orf122	127687	Microarray	1593	EME1	146956	Biotin-Microarrays
518.	TSHZ2	128553	HITS-CLIP	1594	USP25	29761	Biotin-Microarrays
519.	ACVR1C	130399	Luciferase reporter assay//Reporter assay	1595	MIER3	166968	Biotin-Microarrays
520.	CPNE4	131034	Microarray	1596	RMND1	55005	Biotin-Microarrays
521.	GNPDA2	132789	Microarray	1597	PSD3	23362	Biotin-Microarrays
522.	ZNF786	136051	HITS-CLIP	1598	GNAQ	2776	Biotin-Microarrays
523.	MTPN	136319	Microarray	1599	DRAM2	128338	PAR-CLIP
524.	MPLKIP	136647	HITS-CLIP	1600	BACH1	571	HITS-CLIP
525.	PTPDC1	138639	HITS-CLIP	1601	BACH1	571	Biotin-Microarrays
526.	DOCK11	139818	PAR-CLIP	1602	SCAF4	57466	Biotin-Microarrays
527.	BRI3BP	140707	Microarray	1603	FGF18	8817	Biotin-Microarrays
528.	SMCR8	140775	PAR-CLIP	1604	EEF1A1	1915	Biotin-Microarrays
529.	ROMO1	140823	HITS-CLIP	1605	BAG4	9530	Biotin-Microarrays
530.	KDELC2	143888	HITS-CLIP	1606	VPS8	23355	Biotin-Microarrays
531.	PTGR2	145482	HITS-CLIP	1607	EIF4A2	1974	Biotin-Microarrays
532.	RUNDC1	146923	HITS-CLIP	1608	BRPF1	7862	Biotin-Microarrays
533.	BROX	148362	HITS-CLIP	1609	ATP2B2	491	CLEAR-CLIP
534.	TMEM56	148534	HITS-CLIP	1610	FZD1	8321	Biotin-Microarrays
535.	DUSP18	150290	HITS-CLIP	1611	TMEM164	84187	Microarrays
536.	FAM117B	150864	Luciferase reporter assay//qRT-PCR	1612	TAB3	257397	Biotin-Microarrays
537.	TCF23	150921	HITS-CLIP	1613	SKI	6497	Biotin-Microarrays
538.	GPR155	151556	HITS-CLIP	1614	RNF207	388591	Biotin-Microarrays
539.	SGO1	151648	HITS-CLIP	1615	RIBC1	158787	Microarrays

540.	THAP6	152815	HITS-CLIP	1616	CDA	978	Biotin-Microarrays, Microarrays
541.	SLC38A9	153129	HITS-CLIP	1617	PAXBP1	94104	Biotin-Microarrays
542.	METTL27	155368	HITS-CLIP	1618	GART	2618	Biotin-Microarrays
543.	TMEM74	157753	Microarray	1619	SON	6651	Biotin-Microarrays
544.	FAM120A OS	158293	HITS-CLIP	1620	UBE2Z	65264	Biotin-Microarrays
545.	PGBD4	161779	HITS-CLIP	1621	RUNX1	861	Biotin-Microarrays
546.	ZNF320	162967	PAR-CLIP	1622	CTBP1	1487	Biotin-Microarrays
547.	DENND2 C	163259	Microarray	1623	ABR	29	Microarrays
548.	LCA5	167691	Microarray	1624	WDR4	10785	Biotin-Microarrays
549.	FUT11	170384	HITS-CLIP//PAR-CLIP	1625	U2AF1	7307	Biotin-Microarrays
550.	ZNF431	170959	HITS-CLIP	1626	G6PD	2539	Biotin-Microarrays
551.	FAM9B	171483	HITS-CLIP	1627	RRP1	8568	Biotin-Microarrays
552.	AGO3	192669	PAR-CLIP	1628	TRAPPC10	7109	Biotin-Microarrays
553.	DZIP1L	199221	Microarray	1629	DIP2A	23181	Biotin-Microarrays
554.	ALG14	199857	HITS-CLIP	1630	PRMT2	3275	Microarrays
555.	IBA57	200205	HITS-CLIP	1631	RDH13	112724	Biotin-Microarrays
556.	APOBEC3 A	200315	PAR-CLIP	1632	ZER1	10444	Microarrays
557.	TRIM65	201292	Microarray	1633	IL6R	3570	Biotin-Microarrays
558.	ZSCAN4	201516	Microarray	1634	UBQLN4	56893	Biotin-Microarrays
559.	DNAJC18	202052	Microarray	1635	LRRC14	9684	Biotin-Microarrays
560.	VMA21	203547	PAR-CLIP	1636	ZNF333	84449	Biotin-Microarrays
561.	CDY2B	203611	Microarray	1637	SQSTM1	8878	HITS-CLIP
562.	TMEM136	219902	Microarray	1638	SQSTM1	8878	Biotin-Microarrays
563.	ZNF485	220992	HITS-CLIP//PAR-CLIP	1639	NAPEPLD	222236	Biotin-Microarrays
564.	KIF6	221458	HITS-CLIP	1640	SRSF2	6427	HITS-CLIP
565.	AKR7L	246181	HITS-CLIP//PAR-CLIP	1641	RAVER1	125950	Biotin-Microarrays
566.	SLC25A30	253512	Microarray	1642	SENP3	26168	Biotin-Microarrays
567.	GK5	256356	HITS-CLIP	1643	EIF4A1	1973	Biotin-Microarrays
568.	NALCN	259232	Microarray	1644	WDR90	197335	Biotin-Microarrays
569.	FADS6	283985	HITS-CLIP	1645	SYVN1	84447	Biotin-Microarrays
570.	NKPD1	284353	HITS-CLIP	1646	LRP5	4041	Microarrays

571.	ZNF841	284371	HITS-CLIP	1647	SLC25A34	284723	Biotin-Microarrays
572.	FAM19A3	284467	Microarray	1648	RBBP4	5928	Biotin-Microarrays, Microarrays
573.	SEC14L4	284904	PAR-CLIP	1649	CAMK2N1	55450	Biotin-Microarrays
574.	RABL3	285282	HITS-CLIP	1650	MEGF6	1953	Biotin-Microarrays
575.	XKR6	286046	HITS-CLIP	1651	USP1	7398	PAR-CLIP
576.	DPY19L4	286148	HITS-CLIP	1652	FUBP1	8880	Biotin-Microarrays
577.	C14orf39	317761	Microarray	1653	FLVCR1	28982	Biotin-Microarrays
578.	RAB43	339122	PAR-CLIP	1654	RBM15	64783	Biotin-Microarrays
579.	ACER2	340485	PAR-CLIP	1655	DHX57	90957	Biotin-Microarrays
580.	VSIG1	340547	HITS-CLIP	1656	CCNYL1	151195	Microarrays
581.	FMN1	342184	HITS-CLIP	1657	SLC22A15	55356	Biotin-Microarrays
582.	SMTNL2	342527	HITS-CLIP	1658	AZI2	64343	Biotin-Microarrays
583.	MOGAT3	346606	PAR-CLIP	1659	STT3B	201595	Biotin-Microarrays
584.	FAM71F2	346653	HITS-CLIP	1660	IFI16	3428	Biotin-Microarrays
585.	ZNF233	353355	Microarray	1661	GTPBP8	29083	Microarrays
586.	DNAJB13	374407	HITS-CLIP	1662	PTPN13	5783	Biotin-Microarrays
587.	ANKRD36	375248	HITS-CLIP	1663	ATXN7	6314	Biotin-Microarrays
588.	RBM43	375287	PAR-CLIP	1664	TIPARP	25976	Microarrays
589.	C3orf62	375341	HITS-CLIP	1665	SMIM14	201895	Biotin-Microarrays
590.	VWC2	375567	PAR-CLIP	1666	RPP14	11102	Biotin-Microarrays
591.	ZNF322P1	387328	PAR-CLIP	1667	APBB2	323	Biotin-Microarrays
592.	RGS9BP	388531	HITS-CLIP	1668	CRELD1	78987	Biotin-Microarrays
593.	FLG2	388698	HITS-CLIP	1669	U2SURP	23350	Biotin-Microarrays
594.	PLEKHM 3	389072	HITS-CLIP	1670	TTC14	151613	Biotin-Microarrays
595.	IYD	389434	HITS-CLIP	1671	LIPH	200879	Microarrays
596.	FAM102A	399665	Microarray	1672	PBRM1	55193	Biotin-Microarrays
597.	ZNF321P	399669	Microarray	1673	CDC25A	993	Biotin-Microarrays
598.	HACD4	401494	HITS-CLIP	1674	FBXW12	285231	Biotin-Microarrays
599.	BCL2L15	440603	HITS-CLIP	1675	MST1R	4486	Biotin-Microarrays
600.	CISD2	493856	PAR-CLIP	1676	WDR82	80335	Microarrays
601.	AGAP9	642517	HITS-CLIP	1677	TMEM144	55314	Biotin-Microarrays
602.	ARIH2OS	646450	HITS-CLIP	1678	NAA15	80155	Microarrays

603.	ZBTB8A	653121	HITS-CLIP	1679	FAM160A1	729830	HITS-CLIP
604.	POM121L 7P	728418	HITS-CLIP	1680	NIPBL	25836	Biotin-Microarrays
605.	SLC35E2 B	728661	HITS-CLIP	1681	SLC25A46	91137	PAR-CLIP
606.	PRR23A	729627	HITS-CLIP//PAR-CLIP	1682	CMBL	134147	Biotin-Microarrays
607.	ISPD	729920	HITS-CLIP	1683	WDR41	55255	Biotin-Microarrays
608.	LRRC3C	100505591	HITS-CLIP	1684	SCGB3A2	117156	Biotin-Microarrays
609.	ARL17B	100506084	HITS-CLIP	1685	ERAP1	51752	Biotin-Microarrays
610.	ISY1-RAB43	100534599	PAR-CLIP	1686	RICTOR	253260	Biotin-Microarrays
611.	ABL2	27	HITS-CLIP//PAR-CLIP	1687	ANKRA2	57763	Microarrays
612.	AMD1	262	PAR-CLIP	1688	FOXQ1	94234	Microarrays
613.	CKS1B	1163	PAR-CLIP	1689	CREBRF	153222	Microarrays
614.	FCAR	2204	PAR-CLIP	1690	SFXN1	94081	Biotin-Microarrays
615.	FUT1	2523	ELISA//Luciferase reporter assay//qRT-PCR//Western blot	1691	STXBP5	134957	Biotin-Microarrays
616.	GRK4	2868	HITS-CLIP	1692	TRA2A	29896	Biotin-Microarrays
617.	HMGB1	3146	HITS-CLIP	1693	SAP30L	79685	Biotin-Microarrays
618.	ITGAX	3687	PAR-CLIP	1694	ZNF12	7559	Biotin-Microarrays
619.	KARS	3735	HITS-CLIP	1695	CTSB	1508	Biotin-Microarrays
620.	KCNJ6	3763	HITS-CLIP	1696	SPAG11B	10407	Biotin-Microarrays
621.	LETM1	3954	PAR-CLIP	1697	RASEF	158158	Biotin-Microarrays
622.	NQO2	4835	HITS-CLIP	1698	STRBP	55342	Biotin-Microarrays
623.	PFAS	5198	PAR-CLIP	1699	GAPVD1	26130	HITS-CLIP
624.	RAB3B	5865	HITS-CLIP	1700	TRMT10B	158234	Biotin-Microarrays
625.	RAB27A	5873	HITS-CLIP	1701	BRWD3	254065	Microarrays
626.	RAD51	5888	PAR-CLIP	1702	PGM2L1	283209	Biotin-Microarrays, Microarrays
627.	RPL34	6164	HITS-CLIP	1703	ZNF22	7570	Biotin-Microarrays
628.	SHMT1	6470	PAR-CLIP	1704	EML5	161436	Biotin-Microarrays
629.	SOAT1	6646	PAR-CLIP	1705	NEMF	9147	Biotin-Microarrays
630.	WNT2B	7482	HITS-CLIP	1706	ARF6	382	HITS-CLIP
631.	ZNF91	7644	HITS-CLIP//PAR-CLIP	1707	BEND7	222389	Biotin-Microarrays
632.	ALDH5A1	7915	HITS-CLIP	1708	NSD1	64324	Biotin-Microarrays

633.	APOL1	8542	HITS-CLIP	1709	TSC1	7248	Biotin-Microarrays
634.	PLPP3	8613	HITS-CLIP	1710	METTL3	56339	Biotin-Microarrays
635.	LATS1	9113	HITS-CLIP	1711	TC2N	123036	CLEAR-CLIP
636.	SLC28A2	9153	PAR-CLIP	1712	IFI27	3429	Microarrays
637.	SNAP29	9342	HITS-CLIP	1713	NELL1	4745	Microarrays
638.	SEMA3E	9723	HITS-CLIP	1714	KCNC2	3747	CLEAR-CLIP
639.	CCS	9973	HITS-CLIP//PAR-CLIP	1715	TAF1D	79101	Biotin-Microarrays
640.	ZNF275	10838	PAR-CLIP	1716	SPRED1	161742	Luciferase Reporter Assay
641.	ZFP30	22835	HITS-CLIP	1717	CLMP	79827	Microarrays
642.	PLEKHG3	26030	PAR-CLIP	1718	STXBP4	252983	Biotin-Microarrays
643.	MTG2	26164	HITS-CLIP	1719	WBP1L	54838	Biotin-Microarrays
644.	MCAT	27349	Luciferase reporter assay//Next Generation Sequencing (NGS)//qRT-PCR	1720	ANAPC16	119504	Biotin-Microarrays
645.	WDPCP	51057	HITS-CLIP	1721	TRIM44	54765	Microarrays
646.	TAS2R5	54429	HITS-CLIP	1722	TPP1	1200	Biotin-Microarrays
647.	TMEM106 B	54664	HITS-CLIP	1723	NETO1	81832	Biotin-Microarrays
648.	PGPEP1	54858	PAR-CLIP	1724	AKIP1	56672	Biotin-Microarrays
649.	MRM3	55178	HITS-CLIP	1725	ATMIN	23300	Biotin-Microarrays, Microarrays
650.	SVOP	55530	HITS-CLIP	1726	TMX3	54495	Biotin-Microarrays, Microarrays
651.	CABP4	57010	HITS-CLIP	1727	CCDC68	80323	Microarrays
652.	TIGAR	57103	PAR-CLIP	1728	RIMKLB	57494	Biotin-Microarrays
653.	SHROOM 4	57477	HITS-CLIP	1729	HSP90B1	7184	Biotin-Microarrays
654.	FANCM	57697	HITS-CLIP	1730	B2M	567	Biotin-Microarrays
655.	GTDC1	79712	HITS-CLIP	1731	CASC4	113201	Biotin-Microarrays
656.	CLMP	79827	HITS-CLIP	1732	AP1G1	164	Biotin-Microarrays
657.	ZMYM1	79830	HITS-CLIP//PAR-CLIP	1733	SLFN5	162394	Biotin-Microarrays
658.	NOL10	79954	HITS-CLIP	1734	CATSPER2	117155	Biotin-Microarrays
659.	ORAI2	80228	HITS-CLIP	1735	ANPEP	290	Biotin-Microarrays
660.	KREMEN 1	83999	HITS-CLIP	1736	C18orf54	162681	Biotin-Microarrays

661.	MYPN	84665	HITS-CLIP	1737	NAB2	4665	Biotin-Microarrays
662.	PRPF38A	84950	HITS-CLIP//PAR-CLIP	1738	PATL1	219988	Biotin-Microarrays
663.	ZNF682	91120	HITS-CLIP	1739	ACSF2	80221	CLEAR-CLIP
664.	ACBD5	91452	PAR-CLIP	1740	MIDN	90007	Biotin-Microarrays
665.	GBP4	115361	HITS-CLIP	1741	ZNF641	121274	Biotin-Microarrays
666.	LRRC58	116064	HITS-CLIP	1742	TUBA1A	7846	Microarrays
667.	WDR92	116143	HITS-CLIP	1743	PSCA	8000	Biotin-Microarrays
668.	LRG1	116844	HITS-CLIP	1744	TSR1	55720	Biotin-Microarrays
669.	RNF19B	127544	HITS-CLIP	1745	KLK13	26085	Biotin-Microarrays
670.	MIPOL1	145282	HITS-CLIP	1746	ANGPTL4	51129	Biotin-Microarrays, Microarrays
671.	CCBE1	147372	HITS-CLIP	1747	PRDX2	7001	Biotin-Microarrays
672.	DENND5 B	160518	HITS-CLIP	1748	RAB26	25837	Biotin-Microarrays
673.	TPCN2	219931	HITS-CLIP	1749	SRRM2	23524	Biotin-Microarrays
674.	ZDHHC24	254359	PAR-CLIP	1750	SF1	7536	Biotin-Microarrays
675.	PROSER2	254427	PAR-CLIP	1751	SCARA3	51435	Biotin-Microarrays
676.	GATC	283459	HITS-CLIP	1752	SETD5	55209	Biotin-Microarrays
677.	ZNF843	283933	HITS-CLIP	1753	DNAJC7	7266	Biotin-Microarrays
678.	ZNF283	284349	HITS-CLIP	1754	IRF2BP2	359948	HITS-CLIP
679.	ARL10	285598	HITS-CLIP	1755	PXK	54899	Biotin-Microarrays
680.	RBM43	375287	HITS-CLIP	1756	SCN11A	11280	Biotin-Microarrays
681.	ERCC6L2	375748	PAR-CLIP	1757	ATXN2L	11273	Biotin-Microarrays
682.	NOTCH2 NL	388677	HITS-CLIP	1758	GBX2	2637	Microarrays
683.	GTF2H5	404672	HITS-CLIP	1759	ZNF30	90075	Biotin-Microarrays
684.	GXYLT2	727936	HITS-CLIP	1760	IL7R	3575	Biotin-Microarrays
685.	NFYA	4800	Biotin-Microarrays	1761	NPNT	255743	Biotin-Microarrays
686.	CFLAR	8837	Biotin-Microarrays	1762	SEMA4C	54910	Microarrays
687.	TFPI	7035	Biotin-Microarrays, Microarrays	1763	IL12A	3592	Biotin-Microarrays
688.	NDUFAF7	55471	Biotin-Microarrays	1764	MAT2A	4144	HITS-CLIP
689.	MTMR7	9108	Biotin-Microarrays	1765	COL4A3	1285	Biotin-Microarrays
690.	RBM6	10180	Biotin-Microarrays	1766	GOT1L1	137362	Biotin-Microarrays
691.	POLR2J	5439	Biotin-Microarrays	1767	TBC1D10B	26000	Microarrays

692.	FAM214B	80256	Biotin-Microarrays	1768	MMGT1	93380	Microarrays
693.	CROT	54677	Microarrays	1769	ZEB2	9839	Biotin-Microarrays, HITS-CLIP
694.	KMT2E	55904	Biotin-Microarrays	1770	GKN1	56287	Biotin-Microarrays
695.	ZNF195	7748	Biotin-Microarrays	1771	C15orf40	123207	Biotin-Microarrays
696.	MYCBP2	23077	Biotin-Microarrays	1772	HIC2	23119	Biotin-Microarrays
697.	FBXL3	26224	Biotin-Microarrays	1773	ZNF32	7580	Microarrays
698.	ETV1	2115	Biotin-Microarrays	1774	UGP2	7360	Biotin-Microarrays
699.	PHTF2	57157	Biotin-Microarrays	1775	PCDH7	5099	Biotin-Microarrays
700.	MYLIP	29116	Biotin-Microarrays	1776	MUC3A	4584	HITS-CLIP
701.	E2F2	1870	Biotin-Microarrays	1777	KLF13	51621	Biotin-Microarrays
702.	JARID2	3720	Biotin-Microarrays	1778	TMEM154	201799	PAR-CLIP
703.	DLEC1	9940	Biotin-Microarrays	1779	ALCAM	214	Biotin-Microarrays
704.	CYB561	1534	Biotin-Microarrays	1780	HNRNPA3	220988	Biotin-Microarrays
705.	CELSR3	1951	Biotin-Microarrays	1781	RNF150	57484	Biotin-Microarrays
706.	REV3L	5980	Biotin-Microarrays	1782	USP47	55031	Biotin-Microarrays
707.	POMT2	29954	Biotin-Microarrays	1783	FAM161A	84140	Biotin-Microarrays
708.	ZNF207	7756	Biotin-Microarrays	1784	TMED10	10972	Biotin-Microarrays, PAR-CLIP
709.	CD9	928	Biotin-Microarrays	1785	SMAD1	4086	Biotin-Microarrays
710.	IFFO1	25900	Biotin-Microarrays	1786	SP7	121340	Microarrays
711.	NISCH	11188	Biotin-Microarrays	1787	STAT2	6773	Microarrays
712.	SCMH1	22955	PAR-CLIP, Biotin-Microarrays, Microarrays	1788	RNF34	80196	Biotin-Microarrays
713.	MRC2	9902	Biotin-Microarrays	1789	POLH	5429	Biotin-Microarrays
714.	AKAP8L	26993	Biotin-Microarrays	1790	AKAP13	11214	Biotin-Microarrays
715.	PIK3C2A	5286	Biotin-Microarrays	1791	USP32	84669	Biotin-Microarrays
716.	DCN	1634	Biotin-Microarrays	1792	KIAA0232	9778	Biotin-Microarrays
717.	ERCC1	2067	Biotin-Microarrays	1793	TMEM43	79188	Biotin-Microarrays
718.	CLK1	1195	Biotin-Microarrays	1794	OSCAR	126014	Biotin-Microarrays
719.	DNASE1L1	1774	PAR-CLIP	1795	TANC2	26115	Biotin-Microarrays
720.	CAPN1	823	Biotin-Microarrays	1796	PKIA	5569	Biotin-Microarrays
721.	ACPP	55	Microarrays	1797	MTM1	4534	Microarrays
722.	MDH1	4190	Biotin-Microarrays	1798	TRMT61B	55006	PAR-CLIP
723.	ISL1	3670	Biotin-Microarrays	1799	INSR	3643	Biotin-Microarrays

724.	RUFY3	22902	Biotin-Microarrays	1800	C9orf16	79095	Biotin-Microarrays
725.	CXorf56	63932	Biotin-Microarrays, Microarrays	1801	ZNF692	55657	Biotin-Microarrays
726.	TTC27	55622	Biotin-Microarrays	1802	SHCBP1	79801	Microarrays
727.	CPS1	1373	Biotin-Microarrays	1803	CHD7	55636	Biotin-Microarrays
728.	ABCC2	1244	Biotin-Microarrays	1804	ESCO2	157570	Biotin-Microarrays
729.	PHF20	51230	HITS-CLIP, Biotin-Microarrays	1805	ASXL1	171023	Biotin-Microarrays
730.	CD44	960	Biotin-Microarrays	1806	RSL1D1	26156	HITS-CLIP
731.	BTN3A1	11119	Biotin-Microarrays	1807	ZNF274	10782	Biotin-Microarrays
732.	VEZT	55591	Biotin-Microarrays	1808	PIK3CD	5293	Biotin-Microarrays
733.	BCLAF1	9774	Biotin-Microarrays	1809	SPSB1	80176	Biotin-Microarrays
734.	ANK1	286	Biotin-Microarrays	1810	ATF7IP	55729	Biotin-Microarrays
735.	ZCCHC8	55596	Biotin-Microarrays	1811	PWWP2B	170394	Biotin-Microarrays
736.	UBA6	55236	Biotin-Microarrays, PAR-CLIP	1812	MLLT3	4300	Biotin-Microarrays
737.	PEX3	8504	Biotin-Microarrays	1813	JMJD1C	221037	PAR-CLIP, Biotin-Microarrays
738.	CUL3	8452	PAR-CLIP	1814	SMN1	6606	Biotin-Microarrays
739.	BOD1L1	259282	Biotin-Microarrays	1815	NME6	10201	Biotin-Microarrays
740.	TLL1	7092	Biotin-Microarrays	1816	CYCS	54205	Biotin-Microarrays
741.	TRIO	7204	Biotin-Microarrays	1817	TEFM	79736	Biotin-Microarrays
742.	CDH1	999	Microarrays	1818	ISG20	3669	Microarrays
743.	ZFYVE16	9765	Biotin-Microarrays	1819	ID4	3400	Biotin-Microarrays
744.	PARP3	10039	HITS-CLIP	1820	ZNF131	7690	Biotin-Microarrays
745.	MED17	9440	Biotin-Microarrays	1821	TP53RK	112858	Biotin-Microarrays
746.	DCUN1D1	54165	Biotin-Microarrays	1822	MANEA	79694	Biotin-Microarrays
747.	PHKA2	5256	Biotin-Microarrays	1823	AFF1	4299	PAR-CLIP
748.	EPHA3	2042	Biotin-Microarrays	1824	DCP2	167227	Biotin-Microarrays
749.	DSG2	1829	Biotin-Microarrays	1825	RPL38	6169	Biotin-Microarrays
750.	OFD1	8481	Biotin-Microarrays	1826	CES2	8824	Biotin-Microarrays
751.	GPM6B	2824	Biotin-Microarrays	1827	ANKRD13D	338692	Biotin-Microarrays
752.	TPR	7175	Biotin-Microarrays	1828	HECTD4	283450	Biotin-Microarrays
753.	GOPC	57120	Biotin-Microarrays	1829	FAM222B	55731	Biotin-Microarrays
754.	MRPS10	55173	Microarrays	1830	BNC2	54796	PAR-CLIP
755.	VPS13D	55187	Biotin-Microarrays, HITS-CLIP	1831	KDM2A	22992	Biotin-Microarrays
756.	ADAMTS	11174	Biotin-Microarrays	1832	RAPH1	65059	HITS-CLIP

	6						
757.	LTBP1	4052	Biotin-Microarrays	1833	RAPH1	65059	Biotin-Microarrays
758.	NFE2L3	9603	Biotin-Microarrays	1834	VANGL1	81839	Biotin-Microarrays
759.	LIMA1	51474	Biotin-Microarrays	1835	SLC2A14	144195	Biotin-Microarrays
760.	LAMC3	10319	Biotin-Microarrays	1836	MMRN2	79812	Biotin-Microarrays
761.	MPHOSP H9	10198	Biotin-Microarrays	1837	CHD2	1106	Biotin-Microarrays
762.	SIKE1	80143	Biotin-Microarrays	1838	NMNAT1	64802	Biotin-Microarrays
763.	TTC17	55761	Biotin-Microarrays	1839	HEG1	57493	Biotin-Microarrays
764.	FOXN3	1112	Biotin-Microarrays	1840	RNF213	57674	Biotin-Microarrays
765.	THRAP3	9967	Biotin-Microarrays	1841	NET1	10276	PAR-CLIP
766.	SDCCAG8	10806	Biotin-Microarrays	1842	RBM4	5936	Biotin-Microarrays
767.	KIF1B	23095	CLEAR-CLIP	1843	XXYLT1	152002	Biotin-Microarrays, Microarrays
768.	TBC1D22 A	25771	Microarrays	1844	UBXN2A	165324	Biotin-Microarrays
769.	SYNE2	23224	Biotin-Microarrays	1845	FBXO45	200933	Biotin-Microarrays
770.	PLEKHH1	57475	Biotin-Microarrays	1846	C12orf66	144577	Biotin-Microarrays
771.	CHRDL2	25884	Microarrays	1847	ZHX3	23051	Biotin-Microarrays
772.	SZRD1	26099	HITS-CLIP	1848	RPL4	6124	HITS-CLIP
773.	KMT2C	58508	Biotin-Microarrays	1849	ANKRD36C	400986	CLEAR-CLIP
774.	RC3H2	54542	Biotin-Microarrays	1850	AKIRIN1	79647	HITS-CLIP
775.	SOAT1	6646	CLEAR-CLIP, Microarrays	1851	ANGEL2	90806	Biotin-Microarrays
776.	TMCC3	57458	HITS-CLIP, Biotin-Microarrays	1852	FZD4	8322	Microarrays
777.	ZC3H11A	9877	Biotin-Microarrays	1853	GLMN	11146	Biotin-Microarrays
778.	WNK1	65125	Biotin-Microarrays	1854	DHX36	170506	Biotin-Microarrays
779.	CCAR1	55749	Biotin-Microarrays, Microarrays	1855	CADM2	253559	Biotin-Microarrays
780.	QSER1	79832	Biotin-Microarrays	1856	DDIT3	1649	Microarrays
781.	ELMO2	63916	HITS-CLIP	1857	ARHGAP1	392	Microarrays
782.	VMP1	81671	PAR-CLIP	1858	GOLGA8A	23015	Biotin-Microarrays
783.	AHRR	57491	Microarrays	1859	PHYKPL	85007	Biotin-Microarrays
784.	LIMCH1	22998	Biotin-Microarrays, Microarrays	1860	SMAD2	4087	PAR-CLIP
785.	HIPK2	28996	Biotin-Microarrays	1861	CCDC14	64770	Biotin-Microarrays

786.	LPAR2	9170	Biotin-Microarrays	1862	B3GALT6	126792	Biotin-Microarrays
787.	SUGP2	10147	Biotin-Microarrays	1863	JAKMIP2	9832	Biotin-Microarrays
788.	EYA2	2139	Microarrays	1864	HSF5	124535	Biotin-Microarrays
789.	ANKS1A	23294	Microarrays	1865	ENTHD1	150350	Biotin-Microarrays
790.	PKN2	5586	HITS-CLIP	1866	RTTN	25914	Biotin-Microarrays
791.	SPEN	23013	Biotin-Microarrays	1867	SLC35G1	159371	Microarrays
792.	MYLK	4638	Biotin-Microarrays	1868	EID2B	126272	Biotin-Microarrays
793.	CDK13	8621	Biotin-Microarrays	1869	SYNE3	161176	HITS-CLIP
794.	MTHFD2	10797	Microarrays	1870	LPCAT4	254531	Biotin-Microarrays
795.	ASPM	259266	Biotin-Microarrays	1871	SLCO3A1	28232	Biotin-Microarrays
796.	EVI5	7813	Biotin-Microarrays	1872	PRR15	222171	Microarrays
797.	CBFB	865	Microarrays	1873	MYO1D	4642	Biotin-Microarrays
798.	HEATR6	63897	Biotin-Microarrays	1874	CDK5R1	8851	Biotin-Microarrays
799.	PSME4	23198	Biotin-Microarrays	1875	WSB2	55884	Biotin-Microarrays
800.	RORA	6095	Biotin-Microarrays	1876	CHD9	80205	Biotin-Microarrays
801.	NUCB2	4925	Biotin-Microarrays	1877	TGIF1	7050	Biotin-Microarrays
802.	TMEM260	54916	Biotin-Microarrays	1878	RPLP2	6181	Biotin-Microarrays
803.	MNT	4335	Biotin-Microarrays	1879	CSTF2T	23283	Microarrays
804.	ST6GALN AC1	55808	Microarrays	1880	AGTRAP	57085	Microarrays
805.	OSBPL3	26031	Biotin-Microarrays	1881	ZNF518A	9849	Biotin-Microarrays
806.	RAD18	56852	PAR-CLIP	1882	ZNF619	285267	CLEAR-CLIP
807.	ATP2B1	490	Biotin-Microarrays	1883	ZBTB41	360023	PAR-CLIP
808.	MAP4K4	9448	Biotin-Microarrays	1884	DDX10	1662	Biotin-Microarrays
809.	MBD3	53615	Biotin-Microarrays	1885	DALRD3	55152	Biotin-Microarrays
810.	RDH11	51109	Biotin-Microarrays	1886	LCORL	254251	Biotin-Microarrays
811.	LNX1	84708	Biotin-Microarrays	1887	GALNT11	63917	Biotin-Microarrays
812.	AFF4	27125	Biotin-Microarrays	1888	GEN1	348654	Biotin-Microarrays
813.	NDE1	54820	Biotin-Microarrays	1889	DNAJC22	79962	Biotin-Microarrays
814.	ALPK1	80216	Biotin-Microarrays	1890	OTOS	150677	Biotin-Microarrays
815.	ST6GAL1	6480	Microarrays	1891	ZNF713	349075	Biotin-Microarrays
816.	CLNS1A	1207	Biotin-Microarrays	1892	SUZ12	23512	Biotin-Microarrays
817.	CA12	771	Biotin-Microarrays	1893	KCTD12	115207	Biotin-Microarrays
818.	NUAK1	9891	Biotin-Microarrays	1894	ZBTB7A	51341	Biotin-Microarrays

819.	ZNF532	55205	Biotin-Microarrays	1895	AURKB	9212	Biotin-Microarrays
820.	ANO8	57719	Microarrays	1896	FAM133A	286499	Biotin-Microarrays
821.	GTSE1	51512	Biotin-Microarrays	1897	DAND5	199699	Biotin-Microarrays
822.	SEMA3C	10512	Microarrays	1898	PTPN11	5781	Biotin-Microarrays
823.	ZNF638	27332	Biotin-Microarrays	1899	PACS2	23241	Biotin-Microarrays
824.	SLC25A40	55972	Biotin-Microarrays	1900	ARL14	80117	Microarrays
825.	RASAL2	9462	Biotin-Microarrays	1901	FLJ37453	729614	Biotin-Microarrays
826.	MARK3	4140	Biotin-Microarrays	1902	PDXDC1	23042	Biotin-Microarrays
827.	FNDC3B	64778	Biotin-Microarrays	1903	TMEM86B	255043	Biotin-Microarrays
828.	FRYL	285527	Biotin-Microarrays	1904	EXOC3	11336	Biotin-Microarrays
829.	ACTB	60	Biotin-Microarrays	1905	ZNF816	125893	Microarrays
830.	PLD1	5337	Biotin-Microarrays	1906	RNF182	221687	Microarrays
831.	DLG1	1739	Biotin-Microarrays	1907	C3orf22	152065	Biotin-Microarrays
832.	MKRN2	23609	Biotin-Microarrays	1908	HOXC9	3225	Biotin-Microarrays
833.	PLXNA2	5362	Biotin-Microarrays	1909	OXTR	5021	Biotin-Microarrays
834.	ANKRD13A	88455	Biotin-Microarrays	1910	SLC26A11	284129	Biotin-Microarrays
835.	ACACB	32	Biotin-Microarrays	1911	TMEM102	284114	PAR-CLIP
836.	TRAF4	9618	Biotin-Microarrays	1912	SGSH	6448	Biotin-Microarrays
837.	IL4R	3566	Microarrays	1913	GPR135	64582	Biotin-Microarrays
838.	USP33	23032	Biotin-Microarrays	1914	TNFSF15	9966	Biotin-Microarrays
839.	SNRPA	6626	PAR-CLIP	1915	RELL1	768211	Biotin-Microarrays
840.	FGFR1	2260	Biotin-Microarrays	1916	C5orf24	134553	Biotin-Microarrays
841.	MAP2	4133	Biotin-Microarrays	1917	CHST15	51363	Microarrays
842.	PIAS2	9063	Biotin-Microarrays	1918	TNRC18	84629	Biotin-Microarrays
843.	PIK3C3	5289	Biotin-Microarrays	1919	ZNF708	7562	Biotin-Microarrays
844.	CBFA2T2	9139	Biotin-Microarrays	1920	EXT1	2131	Biotin-Microarrays
845.	TP73	7161	Biotin-Microarrays	1921	YBEY	54059	Biotin-Microarrays
846.	XRCC5	7520	Biotin-Microarrays	1922	PLCXD1	55344	Biotin-Microarrays
847.	MKNK1	8569	Biotin-Microarrays	1923	EXOC7	23265	Biotin-Microarrays
848.	TNS1	7145	Biotin-Microarrays	1924	CEP97	79598	Biotin-Microarrays
849.	RAPGEF3	10411	Microarrays	1925	SATB1	6304	Biotin-Microarrays
850.	FDFT1	2222	Biotin-Microarrays	1926	TRAK1	22906	Biotin-Microarrays
851.	OPHN1	4983	Biotin-Microarrays	1927	TTC3	7267	Biotin-Microarrays

852.	RIMS1	22999	Biotin-Microarrays	1928	PAPPA	5069	Biotin-Microarrays
853.	RIF1	55183	Biotin-Microarrays	1929	RPL35A	6165	Biotin-Microarrays
854.	CNOT4	4850	Biotin-Microarrays	1930	EWSR1	2130	Biotin-Microarrays
855.	DLGAP4	22839	Biotin-Microarrays	1931	BCOR	54880	Biotin-Microarrays
856.	CFHR2	3080	Biotin-Microarrays	1932	KIAA2026	158358	Biotin-Microarrays
857.	RSBN1	54665	Biotin-Microarrays	1933	ASB7	140460	Biotin-Microarrays
858.	OSTM1	28962	Biotin-Microarrays	1934	TANGO2	128989	Microarrays
859.	ARG2	384	Microarrays	1935	SFXN4	119559	Biotin-Microarrays
860.	ATP8B1	5205	Biotin-Microarrays	1936	IQGAP3	128239	Biotin-Microarrays
861.	ME2	4200	Microarrays	1937	PTP4A2	8073	Microarrays
862.	CCNT2	905	Biotin-Microarrays	1938	DENND5A	23258	Biotin-Microarrays
863.	COBLL1	22837	Microarrays	1939	EIF3C	8663	Biotin-Microarrays
864.	GSK3B	2932	HITS-CLIP	1940	PCDH9	5101	Biotin-Microarrays
865.	TNPO1	3842	Biotin-Microarrays	1941	POU6F1	5463	Biotin-Microarrays
866.	YTHDC1	91746	Biotin-Microarrays	1942	MAP7D2	256714	CLEAR-CLIP
867.	HAL	3034	Biotin-Microarrays	1943	SS18L1	26039	Biotin-Microarrays
868.	APLP2	334	Biotin-Microarrays	1944	SNN	8303	Biotin-Microarrays
869.	EIF3I	8668	HITS-CLIP	1945	NELL2	4753	Biotin-Microarrays
870.	NCOA1	8648	Biotin-Microarrays	1946	ZBTB40	9923	Biotin-Microarrays
871.	ATRX	546	Biotin-Microarrays	1947	FAM110C	642273	Biotin-Microarrays
872.	MECOM	2122	Biotin-Microarrays	1948	SUMO3	6612	Microarrays
873.	SEH1L	81929	Microarrays	1949	JAG2	3714	Biotin-Microarrays
874.	WDR47	22911	Microarrays	1950	MUC6	4588	HITS-CLIP
875.	ABCB1	5243	Biotin-Microarrays	1951	FAF1	11124	Biotin-Microarrays
876.	ZNF213	7760	PAR-CLIP, Biotin-Microarrays	1952	PRPF39	55015	Biotin-Microarrays
877.	CTTN	2017	Biotin-Microarrays	1953	UBALD2	283991	Biotin-Microarrays
878.	DNAJA1	3301	HITS-CLIP	1954	ARL15	54622	Biotin-Microarrays
879.	SNX10	29887	Microarrays	1955	CDK10	8558	Biotin-Microarrays
880.	RBM22	55696	Biotin-Microarrays	1956	SP140L	93349	Biotin-Microarrays
881.	ZFAND6	54469	Biotin-Microarrays	1957	METTL7A	25840	Biotin-Microarrays
882.	PPEF1	5475	Biotin-Microarrays	1958	IFNLR1	163702	Biotin-Microarrays
883.	HUWE1	10075	CLEAR-CLIP	1959	LMLN	89782	Microarrays
884.	PIGS	94005	Microarrays	1960	BRWD1	54014	Biotin-Microarrays
885.	SF3B2	10992	Biotin-Microarrays	1961	MYBL1	4603	Microarrays

886.	KLHL42	57542	Biotin-Microarrays	1962	ADARB2	105	Biotin-Microarrays
887.	GNAS	2778	Biotin-Microarrays	1963	IFIT1	3434	Microarrays
888.	CNOT3	4849	Biotin-Microarrays	1964	ARL17A	51326	Biotin-Microarrays
889.	DOCK9	23348	Biotin-Microarrays	1965	RNPC3	55599	Biotin-Microarrays
890.	TGDS	23483	Biotin-Microarrays	1966	CCDC84	338657	Biotin-Microarrays
891.	COQ9	57017	Biotin-Microarrays	1967	TOR3A	64222	Biotin-Microarrays, Microarrays
892.	KIZ	55857	Biotin-Microarrays	1968	PPP1CC	5501	Microarrays
893.	ANAPC5	51433	Biotin-Microarrays	1969	GLDN	342035	Biotin-Microarrays
894.	SIRT4	23409	Biotin-Microarrays	1970	PCLO	27445	Biotin-Microarrays
895.	FUS	2521	Biotin-Microarrays	1971	INSIG1	3638	HITS-CLIP
896.	RBM41	55285	Biotin-Microarrays	1972	LYRM7	90624	Microarrays
897.	OTUB2	78990	Biotin-Microarrays	1973	TMEM216	51259	Biotin-Microarrays, Microarrays
898.	NECAP1	25977	Microarrays	1974	TEAD1	7003	Biotin-Microarrays, Microarrays
899.	GPATCH2L	55668	Biotin-Microarrays	1975	FNBP1	23048	Biotin-Microarrays
900.	IRAK3	11213	Biotin-Microarrays	1976	TAF9B	51616	Biotin-Microarrays
901.	SPG21	51324	Biotin-Microarrays	1977	PTMA	5757	Biotin-Microarrays
902.	RAB11FIP3	9727	Biotin-Microarrays	1978	FANCA	2175	Biotin-Microarrays
903.	GLG1	2734	Biotin-Microarrays	1979	SECISBP2	79048	Biotin-Microarrays
904.	RBM27	54439	Biotin-Microarrays	1980	SEMA4D	10507	Microarrays, Biotin-Microarrays
905.	OSBPL8	114882	Microarrays	1981	FANCM	57697	Biotin-Microarrays
906.	LAMB1	3912	Biotin-Microarrays	1982	ZNF70	7621	Biotin-Microarrays
907.	CDV3	55573	Biotin-Microarrays	1983	CARD9	64170	Biotin-Microarrays
908.	SCFD1	23256	Biotin-Microarrays	1984	DMBT1	1755	Microarrays
909.	HNRNPC	3183	Biotin-Microarrays	1985	AGRN	375790	Biotin-Microarrays
910.	GPATCH2	55105	Biotin-Microarrays	1986	ZC3H6	376940	Biotin-Microarrays
911.	SEC22C	9117	PAR-CLIP	1987	DCUN1D3	123879	Microarrays
912.	CBX5	23468	Biotin-Microarrays	1988	AGAP4	119016	Biotin-Microarrays
913.	TMEM38B	55151	Microarrays	1989	ZP3	7784	Microarrays

914.	PTGS1	5742	Biotin-Microarrays	1990	C19orf54	284325	Biotin-Microarrays
915.	CWF19L1	55280	Microarrays	1991	NBR1	4077	Biotin-Microarrays
916.	FKBP5	2289	Microarrays	1992	RALGAPA2	57186	Biotin-Microarrays
917.	EFHC1	114327	Biotin-Microarrays	1993	FAM221A	340277	Biotin-Microarrays
918.	JAK2	3717	Biotin-Microarrays	1994	TMEM201	199953	Biotin-Microarrays
919.	RAB18	22931	Biotin-Microarrays	1995	ZDHHC11	79844	Biotin-Microarrays
920.	PRTFDC1	56952	Microarrays	1996	RPL14	9045	Biotin-Microarrays
921.	HNRNPM	4670	Biotin-Microarrays	1997	TRMT2B	79979	Microarrays
922.	MKNK2	2872	Biotin-Microarrays	1998	ZNF292	23036	Biotin-Microarrays
923.	SLC35E4	339665	PAR-CLIP	1999	ZNF567	163081	Biotin-Microarrays
924.	MFNG	4242	Biotin-Microarrays	2000	ARID2	196528	Biotin-Microarrays
925.	GGA1	26088	Biotin-Microarrays	2001	TMEM63A	9725	Biotin-Microarrays
926.	HIRA	7290	Microarrays	2002	ZNF107	51427	Biotin-Microarrays
927.	HPS4	89781	Biotin-Microarrays	2003	ZNF493	284443	Biotin-Microarrays
928.	DDX17	10521	Biotin-Microarrays	2004	EPHB4	2050	Biotin-Microarrays
929.	CBY1	25776	Microarrays	2005	ZNF124	7678	Biotin-Microarrays
930.	RHBDD3	25807	Biotin-Microarrays	2006	TSC22D2	9819	Biotin-Microarrays
931.	CHKB	1120	Biotin-Microarrays	2007	NCOR2	9612	Biotin-Microarrays
932.	MTMR3	8897	Biotin-Microarrays	2008	LAMA2	3908	Microarrays
933.	PNPLA3	80339	Biotin-Microarrays	2009	XRCC2	7516	Biotin-Microarrays
934.	TNRC6B	23112	Biotin-Microarrays	2010	SLC22A25	387601	Biotin-Microarrays
935.	IFT27	11020	Biotin-Microarrays	2011	RABL6	55684	Biotin-Microarrays
936.	ZC3H7B	23264	Biotin-Microarrays	2012	TRAPPC4	51399	Microarrays
937.	BRD1	23774	Biotin-Microarrays	2013	AMZ2	51321	Microarrays
938.	SOS2	6655	Biotin-Microarrays	2014	NF1	4763	Biotin-Microarrays
939.	SNW1	22938	Biotin-Microarrays	2015	COL27A1	85301	Biotin-Microarrays
940.	DICER1	23405	Biotin-Microarrays	2016	ZNF700	90592	Biotin-Microarrays
941.	PAPLN	89932	Biotin-Microarrays	2017	MVB12B	89853	Biotin-Microarrays
942.	NFKBIA	4792	Biotin-Microarrays	2018	ANKRD36B	57730	Biotin-Microarrays
943.	MYBL2	4605	Microarrays	2019	ARHGEF12	23365	Biotin-Microarrays
944.	NDRG3	57446	Microarrays	2020	SRGAP1	57522	Biotin-Microarrays
945.	PABPC1L	80336	HITS-CLIP, Biotin-Microarrays	2021	FAM3C	10447	Microarrays
946.	STK4	6789	Biotin-Microarrays	2022	CASP4	837	Biotin-Microarrays, Microarrays

947.	RNF24	11237	HITS-CLIP	2023	ZNF100	163227	Biotin-Microarrays
948.	PLCB4	5332	Biotin-Microarrays	2024	PCBP2	5094	Biotin-Microarrays
949.	TM9SF4	9777	Biotin-Microarrays	2025	PGAP1	80055	Biotin-Microarrays
950.	NOP56	10528	Biotin-Microarrays	2026	NOL4L	140688	Biotin-Microarrays, PAR-CLIP
951.	TTI1	9675	Biotin-Microarrays	2027	SLC2A7	155184	CLEAR-CLIP
952.	E2F1	1869	qPCR, Western Blot	2028	ZNF165	7718	Biotin-Microarrays
953.	TNNC2	7125	Microarrays	2029	PELI1	57162	Biotin-Microarrays, PAR-CLIP
954.	SMCHD1	23347	Biotin-Microarrays	2030	ZNF675	171392	Biotin-Microarrays
955.	RIOK3	8780	Biotin-Microarrays	2031	DIO3	1735	Microarrays
956.	MID1	4281	Biotin-Microarrays	2032	IPP	3652	Biotin-Microarrays
957.	MOSPD1	56180	Microarrays	2033	SPN	6693	Biotin-Microarrays
958.	CCDC22	28952	Biotin-Microarrays	2034	MIB2	142678	Biotin-Microarrays
959.	GABRE	2564	Biotin-Microarrays	2035	RAB40C	57799	Biotin-Microarrays
960.	SYTL4	94121	Biotin-Microarrays	2036	PARVA	55742	Biotin-Microarrays
961.	CENPI	2491	Biotin-Microarrays	2037	CCDC180	100499483	Biotin-Microarrays
962.	FNDC3A	22862	Microarrays	2038	ZNF44	51710	Biotin-Microarrays
963.	CDADC1	81602	CLEAR-CLIP	2039	NKIRAS1	28512	Microarrays
964.	STK24	8428	Microarrays	2040	SPG7	6687	Biotin-Microarrays
965.	ARHGEF7	8874	Biotin-Microarrays	2041	MPZL1	9019	Biotin-Microarrays
966.	SUPT20H	55578	Biotin-Microarrays	2042	SNHG12	85028	Biotin-Microarrays
967.	MRPS31	10240	Biotin-Microarrays	2043	TUBA3C	7278	Microarrays
968.	FLT1	2321	Biotin-Microarrays	2044	ZNF273	10793	Biotin-Microarrays
969.	VWA8	23078	Biotin-Microarrays	2045	NUP62CL	54830	Microarrays
970.	MSLN	10232	Biotin-Microarrays	2046	SFI1	9814	Biotin-Microarrays
971.	NFAT5	10725	Biotin-Microarrays, Microarrays	2047	ZNF583	147949	Microarrays
972.	LONP2	83752	Biotin-Microarrays	2048	ZNF28	7576	Biotin-Microarrays
973.	ARL2BP	23568	Microarrays	2049	KCNRG	283518	Biotin-Microarrays
974.	DHODH	1723	Biotin-Microarrays	2050	STK39	27347	Microarrays
975.	PSMD7	5713	Biotin-Microarrays	2051	SLC5A3	6526	Biotin-Microarrays
976.	SETD6	79918	Biotin-Microarrays	2052	CNOT7	29883	Biotin-Microarrays
977.	MLYCD	23417	CLEAR-CLIP	2053	MTOR	2475	qPCR, Western Blot
978.	TAF1C	9013	Biotin-Microarrays	2054	PNP	4860	PAR-CLIP
979.	ZNF500	26048	Biotin-Microarrays	2055	SFT2D1	113402	Microarrays

980.	MTHFSD	64779	Biotin-Microarrays	2056	ZNF277	11179	Biotin-Microarrays
981.	UBE2I	7329	Biotin-Microarrays	2057	LTN1	26046	Biotin-Microarrays
982.	PIEZO1	9780	Biotin-Microarrays	2058	CCDC152	100129792	Biotin-Microarrays
983.	GSPT1	2935	Biotin-Microarrays	2059	SMC5	23137	Biotin-Microarrays
984.	USP31	57478	Biotin-Microarrays	2060	L1CAM	3897	Biotin-Microarrays
985.	TOX3	27324	PAR-CLIP	2061	SREBF2	6721	Biotin-Microarrays
986.	TMC5	79838	Biotin-Microarrays	2062	RPL39	6170	Biotin-Microarrays
987.	KNOP1	400506	Biotin-Microarrays	2063	ATG9A	79065	HITS-CLIP
988.	TMEM87 A	25963	Microarrays	2064	NOS1AP	9722	Biotin-Microarrays
989.	CEP152	22995	Biotin-Microarrays	2065	DMD	1756	Biotin-Microarrays
990.	DTWD1	56986	Biotin-Microarrays	2066	RORB	6096	Biotin-Microarrays
991.	CSPP1	79848	Biotin-Microarrays	2067	SNHG5	387066	Biotin-Microarrays
992.	FZD3	7976	PAR-CLIP	2068	GIGYF2	26058	Biotin-Microarrays
993.	PLAT	5327	Biotin-Microarrays, Microarrays	2069	RUFY2	55680	Biotin-Microarrays
994.	TRPS1	7227	Biotin-Microarrays, PAR-CLIP	2070	BRD2	6046	Biotin-Microarrays
995.	INTS10	55174	Biotin-Microarrays	2071	PBX2	5089	Microarrays
996.	MTMR9	66036	Biotin-Microarrays	2072	MRPL38	64978	Biotin-Microarrays
997.	TNFRSF1 0A	8797	Biotin-Microarrays	2073	GPANK1	7918	Biotin-Microarrays
998.	ERICH1	157697	Biotin-Microarrays	2074	ZNF805	390980	Biotin-Microarrays
999.	ARHGEF1 0	9639	Biotin-Microarrays	2075	TCTN1	79600	Biotin-Microarrays
1000.	KLHDC4	54758	Biotin-Microarrays	2076	PRSS1	5644	Biotin-Microarrays
1001.	MCM4	4173	Microarrays	2077	SPIRE2	84501	Biotin-Microarrays
1002.	ASAH1	427	Microarrays	2078	VIT	5212	Microarrays
1003.	MAN2B1	4125	Biotin-Microarrays	2079	E2F4	1874	PAR-CLIP
1004.	HNRNPL	3191	Biotin-Microarrays	2080	PDE7A	5150	Biotin-Microarrays
1005.	DOT1L	84444	Biotin-Microarrays	2081	IPO7	10527	Microarrays
1006.	MED26	9441	Biotin-Microarrays	2082	TMEM256	254863	Biotin-Microarrays
1007.	CCNE1	898	Biotin-Microarrays	2083	ITPRIPL2	162073	Biotin-Microarrays
1008.	URI1	8725	Biotin-Microarrays	2084	CRYZL1	9946	Biotin-Microarrays
1009.	BCAT2	587	Microarrays	2085	RAB12	201475	Biotin-Microarrays
1010.	MIER2	54531	Biotin-Microarrays	2086	ANKRD28	23243	Biotin-Microarrays

1011.	TNPO2	30000	Biotin-Microarrays	2087	ZNF580	51157	Biotin-Microarrays
1012.	CACNG7	59284	Biotin-Microarrays	2088	ZNF611	81856	Biotin-Microarrays
1013.	PDE4C	5143	Biotin-Microarrays	2089	DENND1B	163486	Biotin-Microarrays
1014.	SCN1B	6324	Biotin-Microarrays	2090	ZNF254	9534	Biotin-Microarrays
1015.	ZNF85	7639	Biotin-Microarrays	2091	GSTM2	2946	Microarrays
1016.	AVL9	23080	Biotin-Microarrays	2092	DNASE1	1773	Biotin-Microarrays
1017.	CFAP69	79846	Biotin-Microarrays	2093	IRF9	10379	HITS-CLIP
1018.	PIK3CG	5294	Luciferase Reporter Assay, qPCR, Western Blot	2094	IRF9	10379	Microarrays
1019.	PON2	5445	Biotin-Microarrays, Microarrays	2095	TAX1BP3	30851	Microarrays
1020.	ITGB8	3696	Biotin-Microarrays	2096	GANC	2595	Biotin-Microarrays
1021.	SSBP1	6742	Biotin-Microarrays	2097	TTLL3	26140	Biotin-Microarrays
1022.	GRB10	2887	Microarrays	2098	C3orf56	285311	Biotin-Microarrays
1023.	ABHD11	83451	Biotin-Microarrays	2099	MIR99AHG	388815	Microarrays
1024.	PTCD1	26024	Biotin-Microarrays	2100	PPP3R1	5534	Microarrays
1025.	ZKSCAN1	7586	PAR-CLIP	2101	PPP2R2A	5520	Biotin-Microarrays, CLEAR-CLIP
1026.	EIF3B	8662	Biotin-Microarrays	2102	CCNL2	81669	Biotin-Microarrays
1027.	USP42	84132	PAR-CLIP, Biotin-Microarrays	2103	EXOSC6	118460	Biotin-Microarrays
1028.	COA1	55744	Biotin-Microarrays	2104	SLC26A6	65010	Biotin-Microarrays
1029.	URGCP	55665	Biotin-Microarrays	2105	RUSC1-AS1	284618	Biotin-Microarrays
1030.	PRKAG2	51422	Biotin-Microarrays	2106	TEX41	401014	Biotin-Microarrays
1031.	LIMK1	3984	Microarrays	2107	SAPCD1	401251	Biotin-Microarrays
1032.	TGFBR1	7046	Biotin-Microarrays	2108	TMA7	51372	Biotin-Microarrays
1033.	SEC61B	10952	Biotin-Microarrays	2109	PPP1R3E	90673	Biotin-Microarrays
1034.	RLN1	6013	Microarrays	2110	OR10G9	219870	CLEAR-CLIP
1035.	RIC1	57589	HITS-CLIP	2111	LRRC37A2	474170	Biotin-Microarrays
1036.	EIF3A	8661	HITS-CLIP	2112	CDRT4	284040	Biotin-Microarrays
1037.	DDX50	79009	Biotin-Microarrays	2113	NSUN6	221078	Biotin-Microarrays
1038.	LZTS2	84445	Biotin-Microarrays	2114	CRCP	27297	Biotin-Microarrays
1039.	SFXN3	81855	Biotin-Microarrays	2115	MICAL3	57553	Biotin-Microarrays
1040.	TNKS2	80351	Biotin-Microarrays	2116	RBM12	10137	Microarrays
1041.	ARHGAP 21	57584	Biotin-Microarrays	2117	N4BP2L2	10443	Biotin-Microarrays

1042.	CPEB3	22849	Microarrays	2118	NEAT1	283131	Biotin-Microarrays
1043.	SMC3	9126	Biotin-Microarrays	2119	PDCD6	10016	Biotin-Microarrays
1044.	GIT1	28964	HITS-CLIP, Biotin-Microarrays, Microarrays	2120	CDK3	1018	Biotin-Microarrays
1045.	RPS6KB1	6198	qPCR, Western Blot	2121	PRKDC	5591	CLEAR-CLIP
1046.	HOXB6	3216	Biotin-Microarrays	2122	PIGY	84992	Biotin-Microarrays
1047.	RAI1	10743	Biotin-Microarrays	2123	EID3	493861	Biotin-Microarrays
1048.	MED31	51003	Biotin-Microarrays	2124	POLR2M	81488	Microarrays
1049.	AKAP10	11216	Biotin-Microarrays	2125	ZNF10	7556	Biotin-Microarrays
1050.	DHX58	79132	Microarrays	2126	CUX1	1523	Biotin-Microarrays
1051.	COL1A1	1277	Microarrays	2127	PRSS58	136541	Biotin-Microarrays
1052.	ABCC3	8714	Biotin-Microarrays	2128	CEP95	90799	Biotin-Microarrays
1053.	LUC7L3	51747	Biotin-Microarrays	2129	ANKRD20A1	84210	Biotin-Microarrays
1054.	PRKAR1A	5573	Biotin-Microarrays	2130	FLJ42627	645644	Biotin-Microarrays
1055.	MMD	23531	Biotin-Microarrays	2131	SRSF8	10929	Biotin-Microarrays
1056.	OCIAD1	54940	Biotin-Microarrays	2132	OTUD7B	56957	Biotin-Microarrays
1057.	AREG	374	Microarrays	2133	RNF115	27246	Biotin-Microarrays
1058.	MFSD10	10227	Microarrays	2134	SEC22B	9554	Microarrays
1059.	GLRB	2743	Biotin-Microarrays	2135	SRGAP2	23380	Biotin-Microarrays
1060.	RAPGEF2	9693	Biotin-Microarrays	2136	POLR2J2	246721	Biotin-Microarrays
1061.	KLF3	51274	Biotin-Microarrays	2137	ZNF224	7767	Biotin-Microarrays
1062.	FOLR1	2348	Biotin-Microarrays	2138	NBPF12	149013	Biotin-Microarrays
1063.	CEP164	22897	Biotin-Microarrays	2139	SLC6A14	11254	Biotin-Microarrays
1064.	CBL	867	Biotin-Microarrays	2140	TMEM185A	84548	Microarrays
1065.	PTPMT1	114971	Microarrays	2141	TAF15	8148	Biotin-Microarrays
1066.	SOX6	55553	Biotin-Microarrays	2142	NBPF10	100132406	Biotin-Microarrays
1067.	NDUFS8	4728	Biotin-Microarrays	2143	CD24	100133941	Microarrays
1068.	EXPH5	23086	Biotin-Microarrays	2144	DCP1A	55802	Microarrays
1069.	CAMKK2	10645	Biotin-Microarrays	2145	CWC25	54883	Biotin-Microarrays
1070.	BCL7A	605	Biotin-Microarrays	2146	ZNF280B	140883	Biotin-Microarrays
1071.	CDKN1B	1027	PAR-CLIP, CLEAR-CLIP	2147	MLLT6	4302	Biotin-Microarrays
1072.	NAA25	80018	Biotin-Microarrays	2148	TIMM22	29928	Microarrays
1073.	SCNN1A	6337	Biotin-Microarrays	2149	CISD3	284106	Biotin-Microarrays
1074.	IL26	55801	Microarrays	2150	MYO19	80179	Biotin-Microarrays

1075.	CHD4	1108	Biotin-Microarrays	2151	C17orf78	284099	Microarrays
1076.	UHRF1BP 1L	23074	Biotin-Microarrays	2152	ACACA	31	Biotin-Microarrays
